# In Vitro Characterization of the Aromatic SAM-Dependent C‑Methyltransferase NapB5

**DOI:** 10.1021/acs.jnatprod.5c01095

**Published:** 2025-10-30

**Authors:** Juliane Breiltgens, Alexandra Paul, Ziruo Zou, Jennifer N. Andexer, Michael Müller

**Affiliations:** Institute of Pharmaceutical Sciences, 9174 University of Freiburg, Albertstr. 25, 79104 Freiburg, Germany

## Abstract

Aromatic polyketide synthase (PKS) products undergo diverse tailoring reactions in the biosynthesis of natural products. *S*-Adenosyl-l-methionine (SAM)-dependent C-methyltransferases (C-MTs) play a key role in this diversification. In the biosynthesis of napyradiomycins, the C-MT NapB5 from *Streptomyces aculeolatus* catalyzes the C2 monomethylation of an 1,3,6,8-tetrahydroxynaphthalene (T_4_HN) building block. Biochemical characterization reveals that NapB5 exhibits chemoselective C-dimethylation activity in vitro, accepting both T_4_HN and its oxidized derivative flaviolin as substrates. Structure-guided mutagenesis and docking studies suggest that precise substrate positioning governs the enzyme’s regio- and chemoselectivity. The proximity between the nucleophilic carbon and the SAM methyl donor is crucial for this selectivity. Furthermore, comparative gene cluster analysis identifies homologous C-MTs in other actinomycetes, underscoring their role in diversifying naphthoquinone-based meroterpenoid natural products.

## Graphical abstract

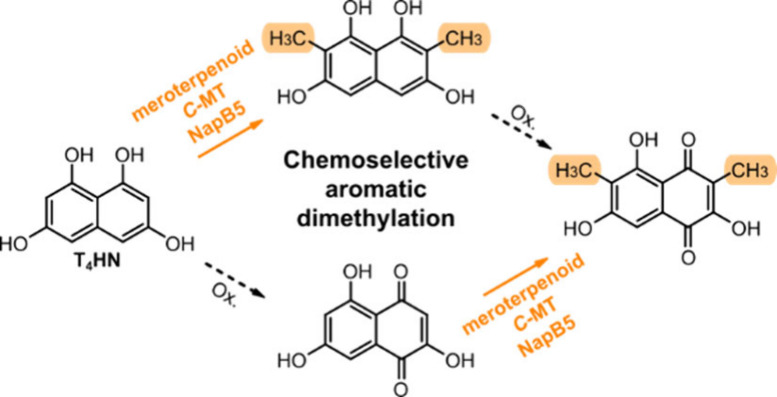

Small molecule C-methyltransferases (C-MTs) are involved in tailoring steps of aromatic building blocks derived from diverse biosynthetic pathways, including the shikimate pathway (e.g., ubiquinone and aromatic amino acids) and the acetate–malonate pathway, among others.^1–4^ C-MTs catalyze the transfer of a methyl group from *S*-adenosyl-l-methionine (SAM, Adomet), nature’s most ubiquitous methyl donor, to a nucleophilic carbon.^5^ In aromatic methylation, electron density at the attacking carbon is often enhanced by an activating hydroxyl group in the *ortho*- or *para*-position. SAM-dependent C-MTs that modify PKS-derived aromatic products are often found in actinomycetes, e.g. in the biosynthesis of antibacterial naphthoquinone-based meroterpenoids.^6^ These highly diverse molecules derive from hybrid biosynthetic origins combining an aromatic polyketide with terpenoid building blocks. Examples of isolated C-methylated naphthoquinone-based meroterpenoids from marine and soil-derived streptomycetes are furanonaphthoquinones,^7^ merochlorins,^8^ furaquinocins,^9,10^ fumaquinones,^11^ naphterpins,^12,13^ and napyradiomycins^14,15^ (Scheme 1). Recently, the Igarashi group discovered C-methylated prenylnaphthoquinones and pyranonaphthoquinones from the rare actinomycete genus *Phytohabitans* (Scheme 1B).^16,17^ The majority of the isolated naphthoquinone-based meroterpenoid natural products are of the napyradiomycin family.^6^ In 2007, Winter et al. reported on the napyradiomycin biosynthetic gene cluster (BGC) in *Streptomyces* sp. CNQ-525 and *Streptomyces aculeolatus*.^18^ The type III PKS product 1,3,6,8-tetrahydroxynaphthalene (T_4_HN) undergoes different tailoring steps including prenylation, halogenation-induced α-hydroxyketone rearrangement, cyclization, and methylation (Scheme 1A).^18–21^ The gene *napB5* was proposed to encode the SAM-dependent MT responsible for C-methylation of the PKS product. In the biosynthesis of the structurally related naphthoquinone-based meroterpenoids furanonaphthoquinone I, furaquinocin A, C, D, I, and J, naphterpin, and merochlorin C and D, the homologous C-MTs Fnq27,^22^ Fur6,^23,24^ NphJ,^25^ and Mcl21,^8^ respectively, are proposed to catalyze similar transformations that result in C3- or C6-methylated naphthoquinone cores. Here, we analyzed the methylation activity of NapB5 in vitro with simplified substrates T_4_HN (**1**) and flaviolin (**2**).

**Scheme 1:**
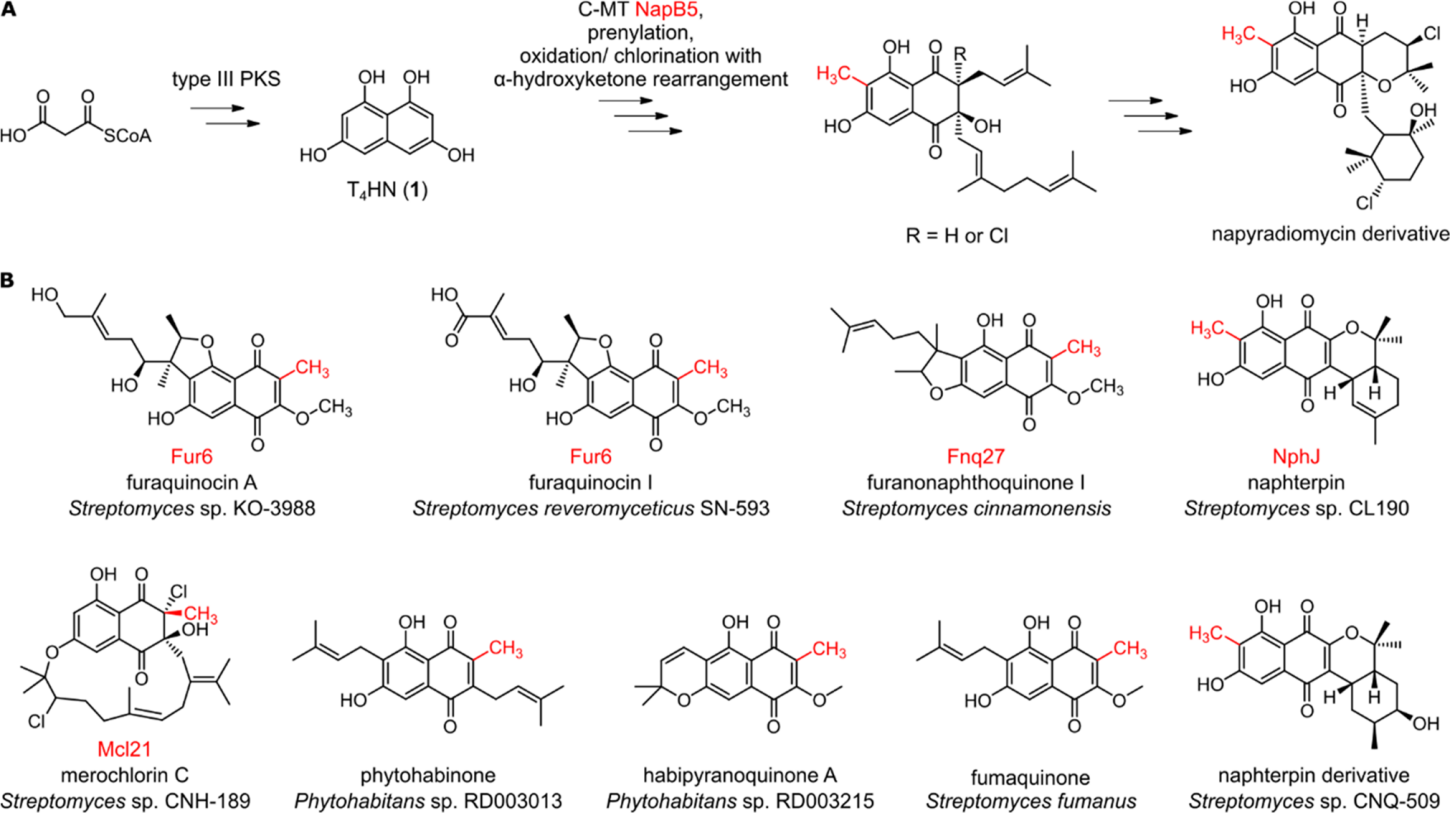
^a^

a (A) Proposed biosynthetic pathway of napyradiomycin in *S. aculeolatus* and *S*. sp. CNQ-525 modified from Winter *et al.*, Bernhardt *et al.*, and Miles *et al*.^18–20^ The relative timing of the C-methylation is unknown. (B) C-Methylated 1,3,6,8-tetrahydroxynaphthalene (T_4_HN, **1**)-derived meroterpenoids with the involved C-MTs given in red (as annotated in literature).

## Results and Discussion

### Biochemical Analysis of NapB5

It has been shown that T_4_HN (**1**) is a likely biosynthetic precursor of napyradiomycins, with prenylation occurring prior to the oxidation, halogenation and cyclization steps.^6^ The relative timing of the C-methylation and the native substrate of NapB5 remains elusive. To investigate the activity of NapB5, the simplified putative substrate **1** and its oxidation product flaviolin (**2**) were tested in vitro. Activity testing was conducted with purified NapB5, which was heterologously produced as a His_6_-tagged fusion protein in *E. coli* (0.2 g·L^–1^). To decelerate spontaneous aerobic oxidation of **1** to **2**, the reducing agent dithiothreitol (DTT) was added to the reaction mixtures (the impact of DTT addition is shown in Figure S4). Additionally, ^13^C-labeled SAM was generated in situ, utilizing a linear enzymatic SAM generation cascade,^26,27^ to differentiate between the mass shift of oxidation (*m*/*z* + 14) and methylation (with ^13^C labeling *m*/*z* + 15) in HPLC-MS analysis (Figure 1A). Both substrates were accepted by NapB5. Surprisingly, NapB5 catalyzes the chemoselective dimethylation of **1** and **2** yielding 2,7-dimethyl-T_4_HN (**4**) and 3,6-dimethylflaviolin (**7**), respectively. To follow the reaction process, the conversion was analyzed at different time points, by quenching the assays after one, eight, and 24 h. After one h, five different products (**3**–**7**) were detected for the conversion of **1** with NapB5 (Figure 1B). The *m*/*z* ratios correspond to monomethylated 2-methyl-T_4_HN (**3**), dimethylated product **4**, and the corresponding oxidation products of **3** and **4** (**5**–**7**). Full conversion of **1** by NapB5 and subsequent aerobic oxidation to **7** is achieved within 24 h. The conversion of **2** results in only two products 3-methylflaviolin (**6**) and **7**, with **6** as main product (Figure 1C bottom). Here, 6-methylflaviolin (**5**) is barely formed, suggesting that for the conversion of **1**, **5** is mainly formed through the aerobic oxidation of **3**. The structures of the stable products **5**, **6**, and **7** were analyzed by comparing the NMR spectra of the extracted activity assays (Figure S11). The ^13^C-labeled methyl groups of **6** and **7** appear as doublets with a heteronuclear coupling (^1^ *J*_CH_) constant of 128 Hz in the ^1^H NMR spectra of extracted activity assays with **2** as the substrate. An additional doublet corresponding to the ^13^C-labeled C6 methyl group of **5** appears in activity assays with **1**. Based on HPLC-MS analysis and structural confirmation of the regio- and chemoselectivity of the methylation reaction by ^1^H NMR, ^13^C NMR, and HSQC analysis (Figure S11–15), we propose the complete reaction process shown in Figure 1A. Comparing the conversion of **2** and **1**, the latter is the favored substrate with complete and faster conversion (partial dimethylation within 1h). These results indicate that the biosynthetic C-methylation step occurs at the level of a precursor derived from **1** rather than at a naphthoquinone derivative.

**Figure 1:**
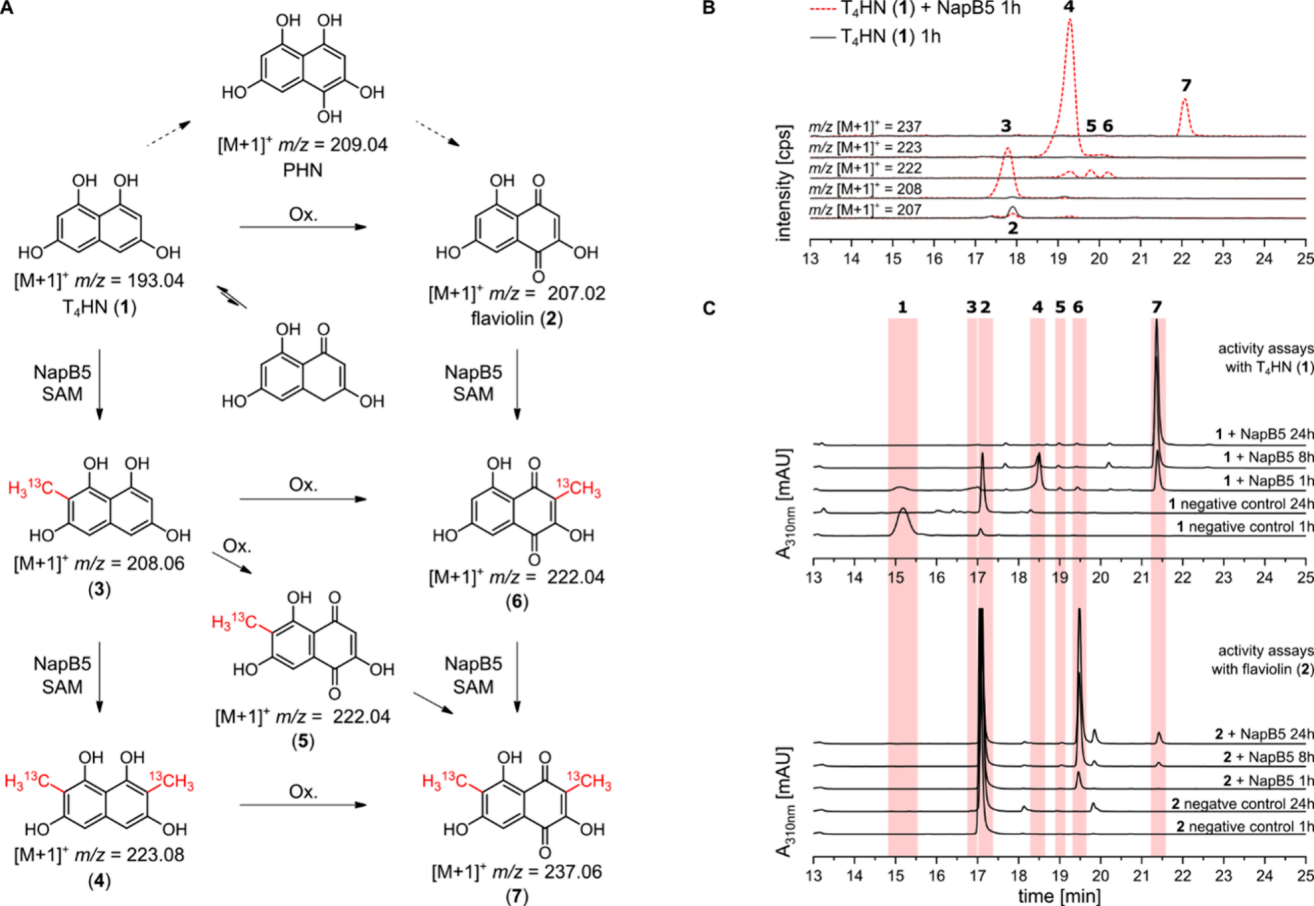
(A) Reaction scheme of T_4_HN (**1**) and flaviolin (**2**) conversion by NapB5. (B) Extracted ion chromatograms of NapB5 activity assays with **1** after 1 h. Red dotted line: assay with NapB5. Black line: negative control without enzyme. (C) HPLC-DAD chromatograms (310 nm) of NapB5 activity assays with **1** and **2**. The assays were performed with purified enzyme.

Interestingly, these results show a discrepancy between the monomethylation activity of NapB5 in the napyradiomycin biosynthetic pathway and its observed dimethylation activity in vitro. In addition, the preferred regiochemistry of in vitro C3-methylation of **2** differs from that of the 6-methylflaviolin core of napyradiomycins. To determine whether the observed dimethylation was caused by an excess of in situ-generated SAM, we repeated the activity assays with **1** using reduced concentrations of the SAM precursors ATP and l-methionine (0.5 mM each). Remarkably, even at equimolar substrate and methyl donor concentrations, the dimethylated product **4** was detectable alongside the monomethylated product **3** within 5 min (Figure S5). The rapid formation of **4** prior to the complete monomethylation of **1** confirms that no excess SAM is required for the dimethylation. This potent dimethylation activity and the regiochemistry observed in vitro suggest that, in the context of the napyradiomycin biosynthesis, the methylation step may occur at a nonsymmetric derivative of **1**, enforcing a selective binding position and leading to regioselective monomethylation by NapB5. Recent studies on the homologous C-methyltransferase Fur6 demonstrated that 1,2,4,5,7-pentahydroxynaphthalene (PHN) serves as putative substrate and undergoes C3 monomethylation.^24,28^ In contrast to NapB5, flaviolin (**2**) is not accepted by Fur6.^24^ The authors propose 8-aminoflaviolin as the common biosynthetic intermediate of T_4_HN-derived meroterpenoids, which has also been isolated from napyradiomycin producer *S*. sp. CNQ-525.^29^ Enzymatic oxidation of **1** to mompain, transaminase catalysis and reductive deamination lead to PHN. Notably, genes encoding the enzymes involved in the formation of 8-aminoflaviolin and the diazo-forming enzyme are conserved in the BGCs of furaquinocin, naphterpin, and furanonaphthoquinone,^24^ and are also present in the BGCs of napyradiomycins (*nap*B1–4). Docking studies of SAM and PHN into a NapB5 AlphaFold2 model show two plausible binding modes in which the methyl group of SAM is aligned with the putative methylation site at C6 (Figure S6C and D). To investigate whether PHN is a substrate of NapB5 yielding **5**, we analyzed the conversion of **2** in the presence of the reducing agent dithionite, as described by Noguchi et al.^24^ The lack of new products alongside products **6** and **7** provides no evidence for conversion of PHN by NapB5 or a change in regioselectivity under the present conditions (Figure S7). In addition to PHN, prenylated precursors could serve as nonsymmetric substrates of NapB5 leading to controlled regioselectivity during biosynthesis. Docking studies of SAM and prenylated T_4_HN derivatives suggest sufficient space to accommodate a prenyl chain at C3 or a geranyl chain at C2, supporting this mechanistic possibility (Figure S6E and F).

### Bioinformatic Analysis of Meroterpenoid C-MTs

To date, seven BGCs of T_4_HN-derived meroterpenoids have been described, and even more natural products from this structural class have been isolated (Figure 1B). To identify homologous C-MTs responsible for modifying the T_4_HN aromatic core, we conducted a comparative genomic analysis of actinomycete strains known to produce these meroterpenoids. Using antiSMASH^30^ for BGC prediction and Clinker^31^ for cluster alignment (Figure S3), we identified that the naphterpin producer *Streptomyces* sp. CNQ-509 contains a BGC encoding naphthoquinone and terpenoid biosynthetic enzymes, including a putative C-MT (WP_253911419.1, *nphJ*; name adapted from C-MT in *S*. sp CL190 in Noguchi 2022 ^25^). In addition, three analyzed *Phytohabitans* strains (Figure S3) harbor closely related BGC, including genes that encode homologous C-MTs (WP_197946283.1, WP_246273153.1, WP_246277640.1). The frequent occurrence of the naphthoquinone C-methylation pattern across T_4_HN-derived meroterpenoids (Figure 1B) together with the identification of C-MT homologues underscores their key role in structural diversification of this natural product family.

The amino acid sequences of the putative C-MTs were analyzed within a phylogenetic framework of known meroterpenoid C-MTs (Figure S1). Particularly, the NapB5 sequences form a separate clade. To further explore the observed sequence variation, we analyzed the multiple sequence alignment (Figure S2) and the NapB5 protein structure predicted by AlphaFold2 ^32^ (Figure 2). Structural analysis revealed that sequence differences extend to key residues within the enzyme’s active site, implying possible functional consequences for the catalytic activity.

**Figure 2:**
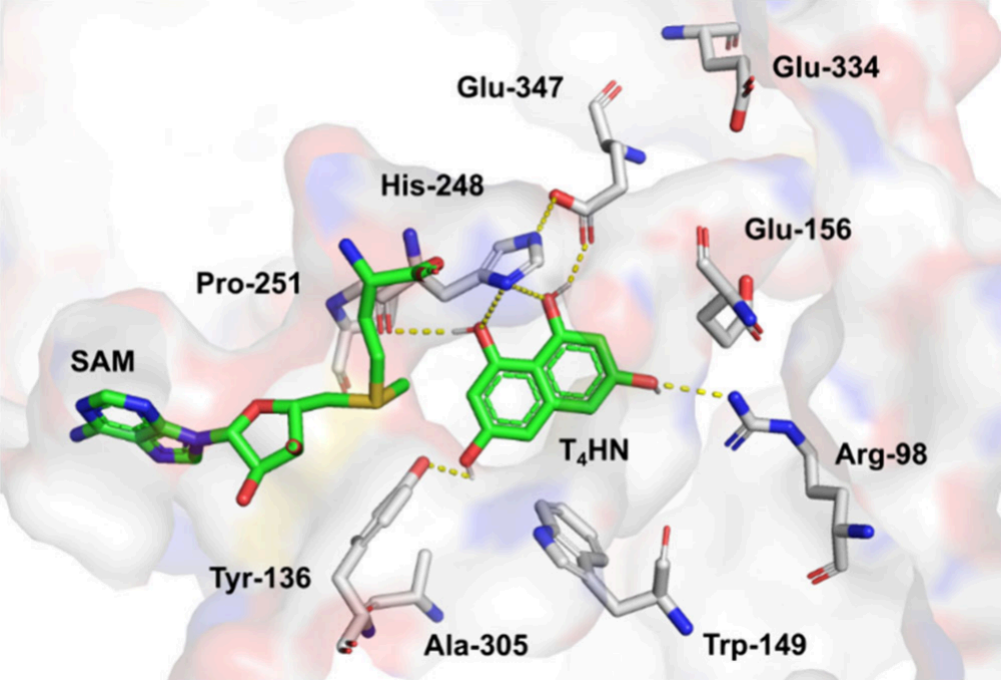
Docking of SAM and T_4_HN (green) into an AlphaFold2 model of NapB5. Putative hydrophilic substrate binding interactions are shown as dashed yellow lines.

### Structural Investigations of NapB5

SAM-dependent MTs share a conserved SAM-binding position on the Rossmann-fold.^5,33,34^ Recognition is mediated in part by residues that interact directly with the carboxypropyl moiety, the ribose hydroxyl groups, and the adenine base of SAM (binding motifs I–III are shown in Figure S2). Superimposition of a NapB5 model with docked SAM onto the SAH-bound crystal structure of Fur6 (PDB: 8HAR) illustrates that the residues involved are highly conserved and adopt a similar SAM-binding conformation (Figure S6B). Additionally, multiple sequence alignment of meroterpenoid C-MTs shows that residues Trp-149, Tyr-136, and His-248 are conserved in the active sites (Figure S2), implying that they play a critical role in catalysis. Further docking studies of SAM and **1** into the NapB5 model reveal key substrate-binding interactions (Figure 2). Tyr-136, His-248, Glu-347, Arg-98, and the backbone of Pro-251 engage in hydrophilic contacts with the substrate’s hydroxyl groups, while three phenylalanine residues (Phe-297, Phe-300, and Phe-301) form a hydrophobic pocket stabilizing the aromatic system (Figure S6A). This binding geometry positions the methyl group of SAM in the proximity of the sp^2^-hybridized C2 of the substrate, which is consistent with the observed regioselectivity in vitro. Mechanistically, deprotonation of the C1/C3 hydroxyl groups likely enhances the nucleophilicity of C2, facilitating methylation. His-248 and Tyr-136 were initially hypothesized to act as catalytic bases, yet single amino acid exchange variants (H248A and Y136F) retain partial activity in lysate assays (H248A: 31–33% conversion; Y136F: 75–80% conversion) and still achieve dimethylation of both **1** and **2** (Figure 3A and B). This suggests that precise substrate positioning and proximity-driven effects rather than strict acid–base catalysis are the dominant factors governing reactivity.

**Figure 3:**
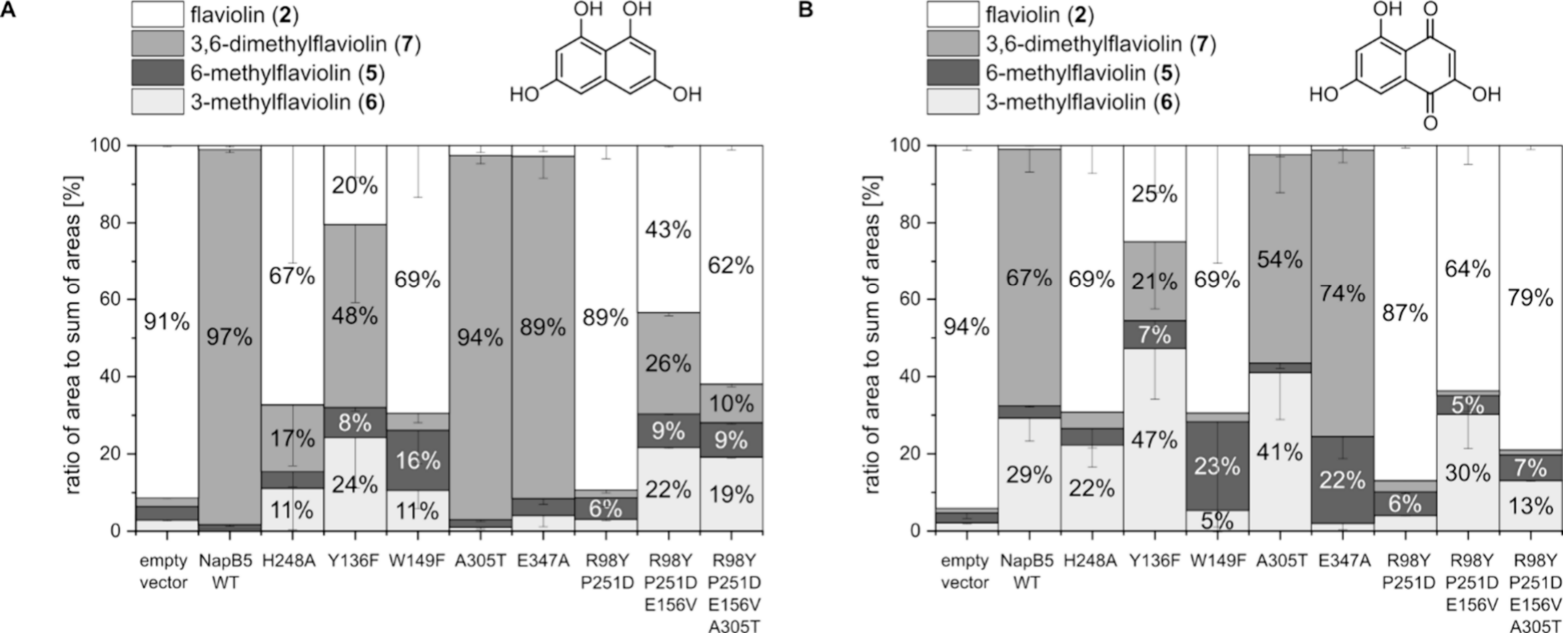
(A) Conversion of T_4_HN (**1**) and (B) flaviolin (**2**) by NapB5 wild-type (WT) and NapB5 variants after 24 h. Activity assays were performed with cell-free lysate. Conversion was calculated based on HPLC-DAD analysis at 310 nm (Figure S8). It should be noted that the positive inductive effect of the methyl groups leads to a bathochromic shift in the absorption spectra (Figure S10). The product formation was interpreted in comparison to wildtype.

Mutation of the conserved Trp-149 leads to a near-complete loss of dimethylation activity (<5%). However, the W149F variant produces 6-methylflaviolin (**5**) (up to 71% in assays with **2**), a product barely observed with the wild-type enzyme (1–3%). This point mutation appears to destabilize the protein, resulting in high variability between biological replicates (e.g., mean of 23.0% with a standard deviation of 28.5 for **5** formation in W149F lysate assays with **2**; Figure 3B). Similarly, the E347A variant also enhances the formation of **5** (Figure 3B). In comparison to W149F, the E347A variant maintains high dimethylation activity with 89% dimethylation of **1** (Figure 3A) and 74% dimethylation of **2** (Figure 3B). The enhanced formation of **5** by the W149F and E347A variants may be due to steric reasons. Replacing Trp-149 with phenylalanine or Glu-347 with alanine likely creates additional space and reduces hydrophilic interactions with the substrate.

Interestingly, NapB5 differs from other homologues, such as Fur6, at five substrate-near residues: Arg-98, Glu-156, Pro-251, Glu-347, and Ala-305 (Figure S2). To assess the catalytic impact of these variations, we generated NapB5 mutants by introducing Fur6-like residues at these positions. Substituting alanine with hydrophilic threonine (A305T) does not affect enzymatic activity. In comparison, multiple variants (R98Y_P251D, R98Y_P251D_E156V, and R98Y_P251D_E156V_A305T) exhibited reduced activity with a complete loss of dimethylation of **2** (Figure 3B). These results underscore the selectivity of aromatic C-MTs, highlighting a clear structure–activity relationship governing their role in specific biosynthetic pathways.

In summary, we characterized the unprecedented dimethylation activity of the *S. aculeolatus* C-MT NapB5 in vitro, demonstrating its ability to selectively mono- and dimethylate aromatic building blocks **1** and **2**. Mechanistically, the nucleophilicity of the acceptor carbon is likely enhanced by phenolate formation via His-248- or Tyr-136-mediated deprotonation. Mutagenesis of active site residues, guided by chemical properties and steric demand as well as rational engineering from enzyme homologues, revealed that precise substrate positioning within the active site is critical for regio- and chemoselective methylation activity, with Trp-149 playing a key structural role. While **1** is the preferred substrate, its symmetry, along with the fact that its monomethylated product **3** is also accepted as the substrate, raises intriguing questions about how regioselectivity is enforced and/or guided in nature. Although the native substrate of NapB5 in the biosynthesis of napyradiomycins remains unknown, structural considerations suggest that prenylation prior to methylation is plausible. Firn and Jones argued that the evolutionary strategy underlying natural product diversity relies on enzymes with inherent promiscuity.^35^ This is illustrated by regio- and substrate-promiscuous aromatic prenyltransferases,^36^ or by angucycline redox modifications, in which enzymes with latent ancestral functions diversify biosynthetic pathways.^37^ Consequently, the shift from a linear interpretation of a biosynthetic pathway to a more holistic perspective may reveal that the relaxed specificity of NapB5 is not a biochemical weakness but may represent a fundamental adaptive feature. Finally, comparative genomic analysis of BGCs for T_4_HN-derived meroterpenoids highlights aromatic C-methylation as a widespread post-PKS tailoring reaction, driving meroterpenoid diversification in actinomycetes. This study advances our understanding of enzymatic aromatic methylation, showcasing the remarkable precision of C-MTs in achieving selective modifications.

## Experimental Section

### General Experimental Procedures

The synthesis of T_4_HN (**1**) and flaviolin (**2**) has been described elsewhere.^38^ High-performance liquid chromatography (HPLC) analyses were performed on a 1260 Infinity II LC system (Agilent Technologies) with a photodiode array detector (DAD). An Agilent Poroshell 120 (RP-C18), 4 × 150 mm, 4.6 μm with a corresponding precolumn from Agilent Technologies served as the stationary phase. The mobile phase with a flow rate of 0.5 mL·min^–1^ was composed of 95% H_2_O with 0.1% formic acid (A) and 5% acetonitrile (B). Gradient: 0–5 min 95% A/5% B, 5–23 min ramp to 5% A/95% B, hold 23–25 min, 25–27 min return to 95% A/5% B, hold 27–30 min. The injection volume was 5 μL and the concentration of the substrates in methanol was 0.5–1 mM. DAD: 310 nm. The same column and gradient were used for HPLC-MS analysis on an Agilent HP 1100 system with an ESI-QTRAP 4500 mass detector (AB Sciex) operated in positive ionization mode. Nuclear Magnetic Resonance Spectroscopy (NMR) spectra were recorded on a DRX 400 spectrometer (Bruker, Rheinstetten, Germany) at 24 °C using 400 MHz for ^1^H NMR spectra and 100.6 MHz for ^13^C NMR. Chemical shifts (δ) are reported in parts per million using the solvent as the internal standard.

### Plasmid Construction

The *S. aculeolatus* gene *napB5* was purchased from Thermo Fisher Scientific as a codon-optimized gene for *E. coli*. The gene was amplified using the oligonucleotides listed in Table S2. All reactions were performed in 20 μL total volume using Phusion High-Fidelity PCR Master Mix with HF Buffer (NEB) following the manufacturer’s instructions. PCR parameters are given in Table S1. The resulting amplicon was ligated to an *EcoR*I-linearized pET28a vector using the In-Fusion Snap Assembly Master Mix (Takara Bio Europe) to yield expression plasmid pET28a::*napB5*.

### Site-Directed Mutagenesis

The target plasmids were amplified in 20 μL total volume using Phusion High-Fidelity PCR Master Mix with HF Buffer (NEB, manufacturer’s instructions), pET28a::*napB5* as template and complementary primers (Table S2) containing the desired mutation. PCR parameters are given in Table S1. Following amplification and *Dpn*I digest of the parental template DNA, Stellar competent cells (Takara Bio Europe) were transformed with 5 μL of the reaction. All constructs were sequenced by Sanger sequencing carried out by Eurofins Genomics Germany GmbH (Ebersberg, Germany).

### Protein Production and Purification

Chemical competent *E. coli* BL21Gold (DE3) cells were transformed with pET28a::*napB5* or the respective plasmids with introduced point-mutations by heat shock. Precultures (5 mL, LB medium) were inoculated with a single colony of the transformed *E. coli* BL21 Gold (DE3) cells and incubated overnight at 37 °C, 170 rpm. Expression cultures (400 mL, LB medium) were inoculated with 2 mL of the preculture and incubated at 37 °C and 170 rpm in baffled 2 L shake flasks. Expression cultures for lysate assays were scaled down to 100 mL in baffled 500 mL shake flasks. Cells were induced with IPTG (0.2 mM final concentration) after the cell density had reached 0.6–0.8 (OD 600 nm). Following incubation for 20 h at 20 °C, expression cultures were harvested by centrifugation (8000 × g, 20 min, 4 °C) with an Avanti J-26S XP centrifuge (JLA-10.500 rotor; Beckmann Coulter, Brea, USA). The cell pellet was resuspended by vortexing in 5 mL of Tris-HCl wash buffer (Tris 50 mM pH 7.5, 10% glycerol, 150 mM NaCl). Cell disruption was performed by sonication with a Sonifier II W–250 (Branson, Danbury, USA). Cell debris was separated by centrifugation and the supernatant was directly used for lysate activity assays (SDS-PAGE Figure S9B) or further purified by Ni-NTA affinity chromatography. The supernatant containing soluble enzyme was loaded on a Ni–NTA agarose column equilibrated with Tris-HCl wash buffer. Unspecifically bound proteins were eluted with a Tris-HCl wash buffer containing 50 mM imidazole. His_6_-tagged NapB5 was eluted with Tris-HCl wash buffer containing 200–400 mM imidazole. Fractions containing the target protein were concentrated to 2.5 mL over a 30 kDa spin filter (Macrosep Advance Centrifugal Device, Pall Corporation, Port Washington, USA) and loaded onto an equilibrated (Tris-HCl wash buffer) PD-10 column (Sephadex G 25 M, Cytiva, USA). The purified protein was eluted with 3.5 mL Tris-HCl wash buffer, analyzed by SDS-PAGE (Figure S9A), and stored as 20% glycerol stock at –20 °C. The production and purification of methionine adenosyltransferase (*Ec*MAT) and methylthioadenosine/SAH nucleosidase (*Ec*MTAN) used in the linear SAM supply cascade were performed as described elsewhere.^26,27^

### In Vitro Activity Assays of NapB5 and Variants

Activity assays were carried out in triplicate in 100 μL reactions. [^13^C-methyl]-SAM was generated in situ from ATP and [^13^C-methyl]-labeled l-methionine by *Ec*MAT. As the byproduct *S*-adenosyl-l-homocysteine (SAH) is an inhibitor for many MTs, *Ec*MTAN was used as a third enzyme in line to irreversibly cleave the SAH yielding *S*-ribosyl-l-homocysteine and adenine.^26,27^ Reactions with purified NapB5 contained 50 mM Tris-HCl buffer pH 7.5, 0.5–1 mM substrate (**1** or **2** in methanol), 50 mM MgCl_2_, 20 mM KCl, 3 mM ATP, 3 mM l-methionine, 10 μM *Ec*MAT, 3 μM *Ec*MTAN, and 30 μM His_6_-NapB5 (1.3 mg·mL^–1^). To decelerate the spontaneous aerobic oxidation of **1**, 1 mM DTT was added to reactions with **1**. For the investigation of PHN conversion, 5 mM dithionite (Na_2_S_2_O_4_) was added to the reactions with **2**. Reactions were incubated 5 min–24 h at 30 °C with shaking (450 rpm), quenched with 10 μL 1 M HCl, and extracted 3× with ethyl acetate. The organic phase was evaporated, and residues were dissolved in 100 μL methanol for HPLC analysis. Negative controls were performed without the addition of His_6_-NapB5. For NMR analysis, 25 reaction extracts were pooled, dried, and dissolved in acetone-*d*_*6*_. Lysate assays were performed with 50 μL of cell-free lysate (estimated final MT concentration: 2 mg·mL^–1^) instead of purified enzyme. Negative controls contained a cell-free lysate of *E. coli* BL21Gold (DE3) cells transformed with the empty vector pET28a.

### Bioinformatics

The multiple sequence alignment of T_4_HN-derived meroterpenoid C-MTs was performed using the MUSCLE^39^ algorithm. Evolutionary analyses were conducted in MEGA11.^40^ The evolutionary history was inferred by using the Maximum Likelihood method and Le_Gascuel_2008 model.^41^ The tree with the highest log likelihood (−5556.91) is shown. Initial tree(s) for the heuristic search were obtained automatically by applying Neighbor-Join and BioNJ algorithms to a matrix of pairwise distances estimated using the JTT model and then selecting the topology with superior log likelihood value. A discrete Gamma distribution was used to model evolutionary rate differences among sites (5 categories (+*G*, parameter = 1.9881)). The tree is drawn to scale with branch lengths measured in the number of substitutions per site (above the branches). This analysis involved 13 amino acid sequences. There were a total of 389 positions in the final data set.

Identification, annotation and analysis of secondary metabolite biosynthesis gene clusters in bacterial genomes was performed by antiSMASH 7.0.^30^ The accession numbers of the used NCBI reference sequences are NZ_AP022871 (*Phytohabitans suffuscus*), NZ_BLPF01000001 (*P. houttuyneae*), NZ_BLPG01000001 (*P. rumicis*), and NZ_CP011492 (*Streptomyces* sp. CNQ-509). Gene cluster comparison was performed with CAGECAT using Clinker.^31,42^

The NapB5 structure model was generated using ColabFold v1.5.5: AlphaFold2.^32,43^ Docking studies were performed with AutoDock4 using the graphical user interface AutoDockTools.^44^

## Supplementary Material



## Data Availability

The antiSMASH annotations of biosynthetic gene clusters as well as the raw data of UV-spectra, HPLC-DAD and HPLC-MS analysis that support the findings of this study are openly available in zenodo.org at 10.5281/zenodo.15673046.

## Notes

Sequences of His_6_-NapB5 and *napB5* gene; ^1^H NMR chemical shifts of **2**, **5**, **6**, and **7**; Supplementary Tables, including PCR conditions, oligonucleotides, and origin of BGCs of T_4_HN-derived meroterpenoids with involved C-MTs; Supplementary Figures, including phylogenetic tree, multiple sequence alignment and BCG analysis of meroterpenoid C-MTs, docking studies with NapB5 model, HPLC-DAD chromatograms of activity assays, SDS-PAGE, UV-spectra of **2**, **6**, and **7**, and NMR spectra. (PDF)
