# Supplementary material for: In Vitro Characterization of the Aromatic SAM-Dependent C‑Methyltransferase NapB5
Source: J Nat Prod. 2025 Oct 30;88(11):2750–6. doi: 10.1021/acs.jnatprod.5c01095 (PMC12670698; doi:10.1021/acs.jnatprod.5c01095)
Supplement: Supplementary file 1 [file np5c01095_si_001.pdf]

# Supporting Information

# In Vitro Characterization of the Aromatic SAM-dependent C-Methyltransferase NapB5

Juliane Breiltgens,[a] Alexandra Paul,[a] Ziruo Zou,[a] Jennifer N. Andexer,[a] and Michael Müller*[a]

[a] Institute of Pharmaceutical Sciences, University of Freiburg, Albertstr. 25, 79104 Freiburg, Germany

michael.mueller@pharmazie.uni-freiburg.de

**1. Sequences of His$_6$-NapB5 and *napB5*** .......... 3

**2. $^1$H NMR chemical shifts of 2, 5, 6, and 7** .......... 3

**3. Supplementary Tables** .......... 4

**Table S1:** PCR conditions. .......... 4
**Table S2:** Oligonucleotides used for gene amplification and site-directed mutagenesis. .......... 4
**Table S3:** Origin of biosynthetic gene clusters of T$_4$HN-derived meroterpenoids with involved C-MTs. .......... 4

**4. Supplementary Figures** .......... 5

**Figure S1:** Phylogenetic analysis of C-MTs from T$_4$HN-derived meroterpenoid biosynthesis. The tree was rooted on the outgroup, which includes three sequences of other aromatic C-MTs from actinomycetes: SfmM2 (ABI22137.1) from *Streptomyces lavendulae*, NovO (Q9L9F3.2) from *Streptomyces niveus*, and OxyF (AAZ78330.1) from *Streptomyces rimosus*. .......... 5
**Figure S2:** Multiple sequence alignment of C-MTs from T$_4$HN-derived meroterpenoid biosynthesis. Putative residues of the active sites are highlighted in red. Conserved SAM-binding motifs I–III are highlighted in blue. .......... 5
**Figure S3:** Biosynthetic gene cluster comparison of T$_4$HN-derived meroterpenoids from ascomycetes. Putative C-MT genes are highlighted in magenta. .......... 6
**Figure S4**: Comparison of HPLC-DAD chromatograms (310 nm) of NapB5 activity assays with **1**, with and without DTT, and **2**. The non-oxidized products **3** and **4** are only detectable when DTT is present. In the absence of DTT, **1** rapidly oxidizes to **2** leading to the main product **6** of **2** conversion. The assays were performed with purified enzyme. .......... 7
**Figure S5**: HPLC-DAD chromatograms (310 nm) of NapB5 activity assays with T$_4$HN (**1**) and SAM precursors (ATP + L-methionine) at equimolar concentrations (0.5 mM each) extracted after 5, 15, and 30 minutes. .......... 7
**Figure S6:** Docking studies of NapB5 model. **A**) Docking of SAM and T$_4$HN (**1**). **B**) Comparison of SAM binding sites by superposition of NapB5 model docked with SAM (grey) onto the crystal structure of SAH-bound Fur6 (red, PDB: 8HAR). **C**) Docking of SAM and PHN (Conformation 1). **D**) Docking of SAM and PHN (Conformation 2). **E**) Docking of SAM and 2-prenyl-T$_4$HN. **F**) Docking of SAM and 4-geranyl-T$_4$HN, which is a proposed intermediate before C4-to-C3 α-hydroxyketone rearrangement of the geranyl moiety.[1] .......... 8

**Figure S7**: **A**) Reduction of flaviolin (**2**) to PHN in the presence of dithionite and conversion of flaviolin by NapB5. **B**) HPLC-DAD chromatograms (310 nm) of NapB5 activity assays with **2** and dithionite extracted after 1 or 24 h. ....... 9
**Figure S8**: HPLC-DAD chromatograms (310 nm) of NapB5 wildtype and NapB5 variants activity assays with flaviolin (**2**) (**A**) and $T_4HN$ (**1**) (**B**) after 24h. The assays were performed with cell-free lysate of E. coli BL21Gold (DE3) cells transformed with the respective constructs. Cell-free lysate of E. coli BL21Gold (DE3) cells transformed with the empty vector pET28a was used for the negative control. Note that in cell-free lysate assays, $T_4HN$ (**1**) and its methylation products (**3** and **4**) undergo complete oxidation within 24 h. ....... 9
**Figure S9: A**) SDS-PAGE of $His_6$-NapB5 (43.2 kDa) Ni-NTA purification. Ladder: NEB Color Prestained Protein Standard. Fraction 1: cell pellet. 2: cell-free lysate. 3: 50 mM imidazole wash step. 4: 250 mM imidazole elution step. 5: desalted fraction. **B**) SDS-PAGE of cell-free lysate of $His_6$-NapB5 wildtype and variants (43.2 kDa). Fraction 1: E. coli BL21 Gold (DE3) cells transformed with pET28a empty vector. 2: NapB5 wildtype. 3: H248A. 4: Y136F. 5: W149F. 6: E347A. 7: A305T. 8 R98Y_P251D. 9: R98Y_P251D_E156V. 10: R98Y_P251D_E156V_A305T. The Coomassie blue staining shows comparable protein concentrations in wildtype and variant lysates. ....... 10
**Figure S10: A**) Extracted UV-spectra from HPLC-DAD peaks of flaviolin (**2**), 3-methylflaviolin (**6**), and 3,6-dimethylflaviolin (**7**). As a compromise for all assays, a wavelength of 310 nm (dashed line) was chosen to minimize the influence of the bathochromic shift on product formation calculations. **B**) Colors of NapB5 activity assays with **2**. ....... 10
🡡**Figure S11:** Comparison of $^1H$ NMR spectra (acetone-$d_6$) of extracted NapB5 activity assays of $T_4HN$ (**1**) or flaviolin (**2**) after 24h (trace c and a) and respective negative controls without enzyme (trace d and b). The signals corresponding to the respective compounds are color-coded. The protons of the $^{13}C$-labeled methyl groups appear as doublets with a heteronuclear coupling ($^1J_{CH}$) constant of 128 Hz. In NapB5 activity assays with **2**, signals of the main product **6** are highlighted in purple. These signals can also be found with lower intensity in assays with **1**. The main product of **1** conversion is **7** (blue) with the two methyl groups visible as doublets (128 Hz each) at 1.99 and 2.12 ppm, respectively. These signals can also be found with lower intensity in assays with **2**. Signals corresponding to **5** (orange) are only visible in assays with **1**. The doublet of the C6-methyl group is overlayed by the C6-methyl group of **7** (2.13 ppm for **5** and 2.12 ppm for **7**), but the aromatic protons of **5** are visible as singlets at 6.10 and 7.18 ppm, respectively. ....... 12
**Figure S12:** $^{13}C$ NMR spectra (acetone-$d_6$) of extracted NapB5 activity assays with $T_4HN$ (**1**) after 1 h and 24 h, with flaviolin (**2**) after 24 h, and respective negative controls without enzyme. Due to low substrate concentrations, only $^{13}C$-labeled methyl signals are visible. After 24 h, the $T_4HN$ (**1**) methylation products **3** and **4** are oxidized to **5**, **6**, and **7** with a signal at 7.44 ppm for the C6-methyl group for **5** and **7**, and 7.12 ppm for C3-methyl group for **6** and **7**. In spectra of 1h activity assays with **1** an additional signal at 6.77 ppm arises for the C2-methyl of **3** and at 6.20 ppm for the methyl group of the respective keto tautomer (**3a**). The small signals, marked with asterisks, may represent the methyl groups of the enol and keto tautomer of **4**. The signal at 7.75 ppm in the NapB5 activity assays with $T_4HN$ (**1**) after 1 h is not assigned because it does not clearly correlate with the $^1H$ spectrum in the HSQC spectrum (see Figure S10). ....... 12
**Figure S13:** HSQC spectrum (acetone-$d_6$) of extracted NapB5 activity assay with $T_4HN$ (**1**) after 1 h. The $^{13}C$ signals of the labeled methyl groups correlate with the $^1J_{CH}$ doublets of the $^1H$ spectrum. ....... 13
**Figure S14:** $^1H$ NMR spectrum (acetone-$d_6$) of extracted NapB5 assays with $T_4HN$ (**1**) after 24 h. ....... 14
**Figure S15:** $^1H$ NMR spectrum (acetone-$d_6$) of extracted NapB5 assays with flaviolin (**2**) after 24 h. ....... 15

## 1. Sequences of His$_6$-NapB5 and *napB5*

### Protein sequence of His$_6$-NapB5 (43.2 kDa)

MGSSHHHHHHSSGLVPRGSHMASMTGGQQMGRGSELLTPEATFTRFREYMVGPSRFMSLLSCFELGVIDNLRDTPGMTAAELGDAVGAKPDAV
EQLLLLLVKESFLAYDEDTGGYSLDALADIAEDDLRQVLARMEMIKVVTLRQLFYLTDSVRTGTTVGLNKLYGHEGNLYDALAEHKDLREPWARLMN
GETAHIDPWFFDNIDVPPGSNVLDLAGNTGLGAIHTCKLKASPGLRVTTFDLPEKEEECLANFRSHGVEEHCSFIGGDVFEEVPKGFDVVLIKHFLPMF
DKSDVFKILEGVNRSMDVGGQVHLLVPVFPENIKDSDNYTVDFFPSFFIGCAMGQGGAQKMSTWQKWLEECGFTVTKAIAEDPADMLPHALPVE
AVLSATKRAG

### Nucleotide sequence of *napB5* (codon optimized for *E. coli*)

CTGACACCGGAAGCAACCTTTACACGTTTTCGTGAATATATGGTTGGTCCGAGCCGTTTTATGAGCCTGCTGAGCTGTTTTGAACTGGGTGTTA
TTGATAATCTGCGTGATACACCGGGTATGACCGCAGCAGAACTGGGAGATGCAGTTGGTGCAAAACCGGATGCCGTTGAACAACTGCTGCTG
CTGTTAGTTAAAGAAAGTTTTCTGGCCTATGATGAAGATACCGGTGGTTATAGCCTGGATGCACTGGCAGATATTGCAGAAGATGATCTGCGT
CAGGTTCTGGCACGTATGGAAATGATTAAAGTTGTTACCCTGCGCCAGCTGTTTTATCTGACCGATAGCGTTCGTACCGGCACCACCGTTGGTC
TGAATAAACTGTATGGTCATGAAGGCAATCTGTATGATGCCCTGGCCGAACATAAAGATCTGCGCGAACCGTGGGCACGTCTGATGAATGGT
GAAACCGCACATATTGATCCGTGGTTTTTCGATAATATTGATGTTCCGCCTGGTAGCAATGTTCTGGATCTGGCAGGTAATACCGGTCTGGGTG
CAATTCATACCTGTAAACTGAAAGCAAGTCCGGGTCTGCGTGTTACCACCTTTGATCTGCCGGAAAAAGAAGAAGAATGTCTGGCAAATTTTC
GTAGCCATGGTGTTGAAGAACACTGCAGCTTTATTGGTGGTGATGTTTTTGAAGAAGTGCCGAAAGGTTTTGATGTGGTGCTGATTAAACATT
TCCTGCCGATGTTTGATAAAAGCGACGTGTTTAAAATCCTGGAAGGTGTTAATCGTAGTATGGATGTTGGTGGTCAGGTTCATCTGCTGGTTCC
GGTTTTTCCTGAAAACATTAAAGATAGCGATAACTATACCGTGGATTTCTTTCCGAGCTTTTTCATTGGTTGTGCAATGGGTCAAGGTGGTGCA
CAGAAAATGAGCACCTGGCAGAAATGGCTGGAAGAATGTGGTTTTACCGTTACCAAAGCAATTGCCGAAGATCCTGCAGATATGCTGCCGCA
TGCACTGCCGGTTGAAGCAGTTCTGAGCGCAACCAAACGTGCAGGTTAA

## 2. $^1$H NMR chemical shifts of 2, 5, 6, and 7

### flaviolin (2)

$^1$H NMR (400 MHz, acetone-$d_6$): δ 6.13 (s, 1 H, H-3), 6.63 (d, $^4J$ = 2.25 Hz, 1 H, H-6), 7.10 (d, $^4J$ = 2.25 Hz, 1 H, H-8), 12.56 (s, 1 H, OH).

### 3-[$^{13}$C]methylflaviolin (6)

$^1$H NMR (400 MHz, acetone-$d_6$): δ 2.01 (d, $^1J_{CH}$ = 128 Hz, 3 H, 3-$^{13}CH_3$), 6.61 (d, $^4J$ = 2.25 Hz, 1 H, H-6), 7.08 (d, $^4J$ = 2.25 Hz, 1 H, H-8), 12.64 (s, 1 H, OH).

### 6-[$^{13}$C]methylflaviolin (5)

$^1$H NMR (400 MHz, acetone-$d_6$): δ 2.13 (d, $^1J_{CH}$ = 128 Hz, 3 H, 6-$^{13}CH_3$), 6.10 (s, 1 H, H-3), 7.18 (s, 1 H, H-8), 12.93 (s, 1 H, OH).

### 3,6-[$^{13}$C]dimethylflaviolin (7)

$^1$H NMR (400 MHz, acetone-$d_6$): δ 1.99 (d, $^1J_{CH}$ = 128 Hz, 3 H, 3-$^{13}CH_3$), 2.12 (d, $^1J_{CH}$ = 128 Hz, 3 H, 6-$^{13}CH_3$), 7.16 (s, 1 H, H-8).

## 3. Supplementary Tables

**Table S1:** PCR conditions.

| Experiment | Initial denaturation | Thermal cycling | Final elongation |
|---|---|---|---|
| Gene amplification | 98 °C for 60 s | 30 cycles of 98 °C for 10 s, 60 °C for 30 s, 72 °C for 50 s | 72 °C for 60 s |
| Site-directed mutagenesis | 98 °C for 60 s | 30 cycles of 98 °C for 30 s, 60 °C for 30 s, 72 °C for 360 s | 72 °C for 60 s |

**Table S2:** Oligonucleotides used for gene amplification and site-directed mutagenesis.

| Oligonucleotide | Sequence | Target construct |
|---|---|---|
| napB5_inf_for | TCGCGGATCCGAATTACTGACACCGGAAGCA | pET28a::*napB5* |
| napB5_inf_rev | CGGAGCTCGAATTTTATTAACCTGCACGTTTG | |
| napB5_Y136F_for | GGCAATCTGTTTGATGCCCTGGCCGAACATAAAG | pET28a::*napB5*_ Y136F |
| napB5_Y136F_rev | CAGGGCATCAAACAGATTGCCTTCATGACCATAC | |
| napB5_P251D_for | CATTTCCTGGACATGTTTGATAAAAGCGACG | pET28a::*napB5*_ R98Y_P251D; pET28a::*napB5*_R98Y_P251D_E156V; pET28a::*napB5*_ R98Y_P251D_E156V_A305T |
| napB5_P251D_rev | CAAACATGTCCAGGAAATGTTTAATCAGCACC | |
| napB5_R98Y_for | GGTTCTGGCATATATGGAAATGATTAAAGTTGTTACCC | |
| napB5_R98Y_rev | CATTTCCATATATGCCAGAACCTGACGCAG | |
| napB5_E156V_for | GAATGGTGTTACCGCACATATTGATCCGTG | |
| napB5_E156V_rev | GTGCGGTAACACCATTCATCAGACGTGCCC | |
| napB5_H248A_for | GCTGATTAAAGCATTCCTGCCGATGTTTG | pET28a::*napB5*_H248A |
| napB5_H248A_rev | CGGCAGGAATGCTTTAATCAGCACCACATC | |
| napB5_W149F_for | GCGAACCGTTCGCACGTCTGATGAATGGTG | pET28a::*napB5*_W149F |
| napB5_W149F_rev | CAGACGTGCGAACGGTTCGCGCAGATCTTTATG | |
| napB5_A305T_for | CATTGGTTGTACCATGGGTCAAGGTGGTGCAC | pET28a::*napB5*_A305T; pET28a::*napB5*_ R98Y_P251D_E156V_A305T |
| napB5_A305T_rev | CTTGACCCATCGTACAACCAATGAAAAAGC | |
| napB5_E347A_for | CTGCCGGTTGCAGCAGTTCTGAGCGCAAC | pET28a::*napB5*_E347A |
| napB5_E347A_rev | CAGAACTGCTGCAACCGGCAGTGCATGCG | |

**Table S3:** Origin of biosynthetic gene clusters of $T_4$HN-derived meroterpenoids with involved C-MTs.

| Natural product | Strain of origin | C-MT | Literature |
|---|---|---|---|
| napyradiomycin analogs | *Streptomyces* sp. CNQ-525 | NapB5 | Winter *et al*. 2007[1] |
| napyradiomycin analogs | *Streptomyces aculeolatus* | NapB5 | Winter *et al*. 2007[1] |
| furaquinocin I/J | *Streptomyces reveromyceticus* SN-593 | Fur6 | Panthee *et al*. 2011[2] |
| furaquinocin A/C/D | *Streptomyces* sp. KO-3988 | Fur6 | Kawasaki *et* al. 2006[3] |
| merochlorin C/D | *Streptomyces* sp. CNH189 | Mcl21 | Kaysser *et al*. 2012[4] |
| furanonaphthoquinone I | *Streptomyces cinnamonensis* DSM 1042 (*S. virginiae*) | Fnq27 | Haagen *et al*. 2006[5] |
| phytohabinone | *Phytohabitans suffuscus* NBRC 105367 | WP_197946283.1 | This study |
| phytohabinone | *Phytohabitans houttuyneae* NBRC 108639 | WP_ 246273153.1 | This study |
| phytohabinone | *Phytohabitans rumicis* NBRC 108638 | WP_246277640.1 | This study |
| naphterpin analog | *Streptomyces* sp. CNQ-509 | WP_253911419.1 | This study |
| naphterpin | *Streptomyces* sp. CL190 | NphJ | Kuzuyama *et al*. 2005[6] Noguchi 2022[7] |

## 4. Supplementary Figures

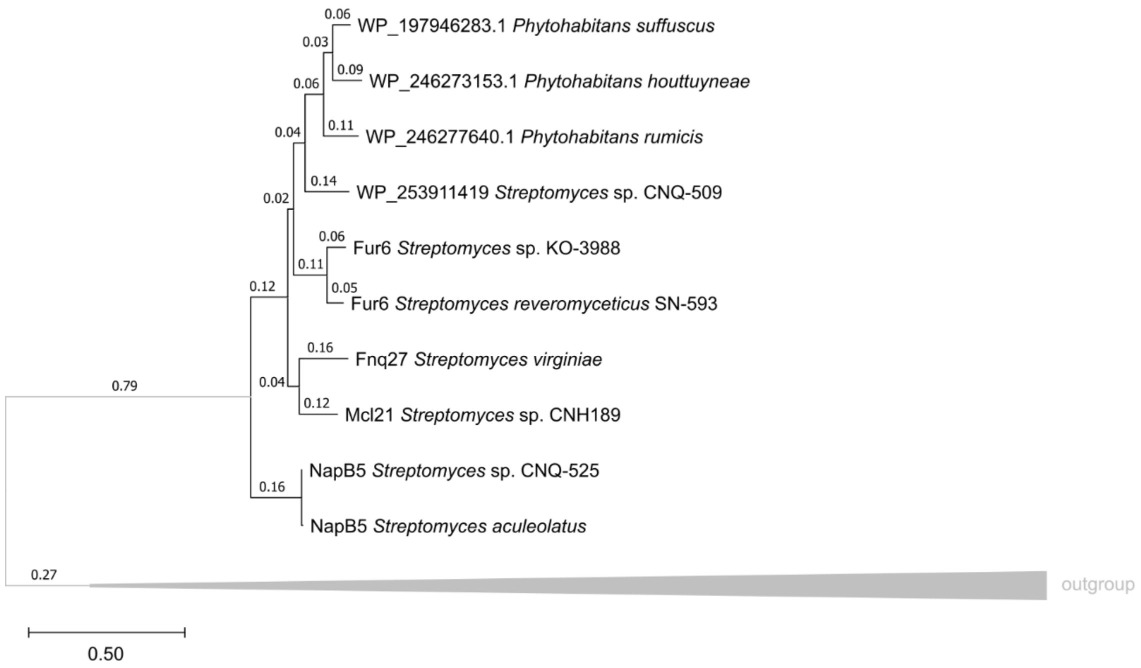


**Figure S1:** Phylogenetic analysis of C-MTs from $T_4HN$-derived meroterpenoid biosynthesis. The tree was rooted on the outgroup, which includes three sequences of other aromatic C-MTs from actinomycetes: SfmM2 (ABI22137.1) from *Streptomyces lavendulae*, NovO (Q9L9F3.2) from *Streptomyces niveus*, and OxyF (AAZ78330.1) from *Streptomyces rimosus*.

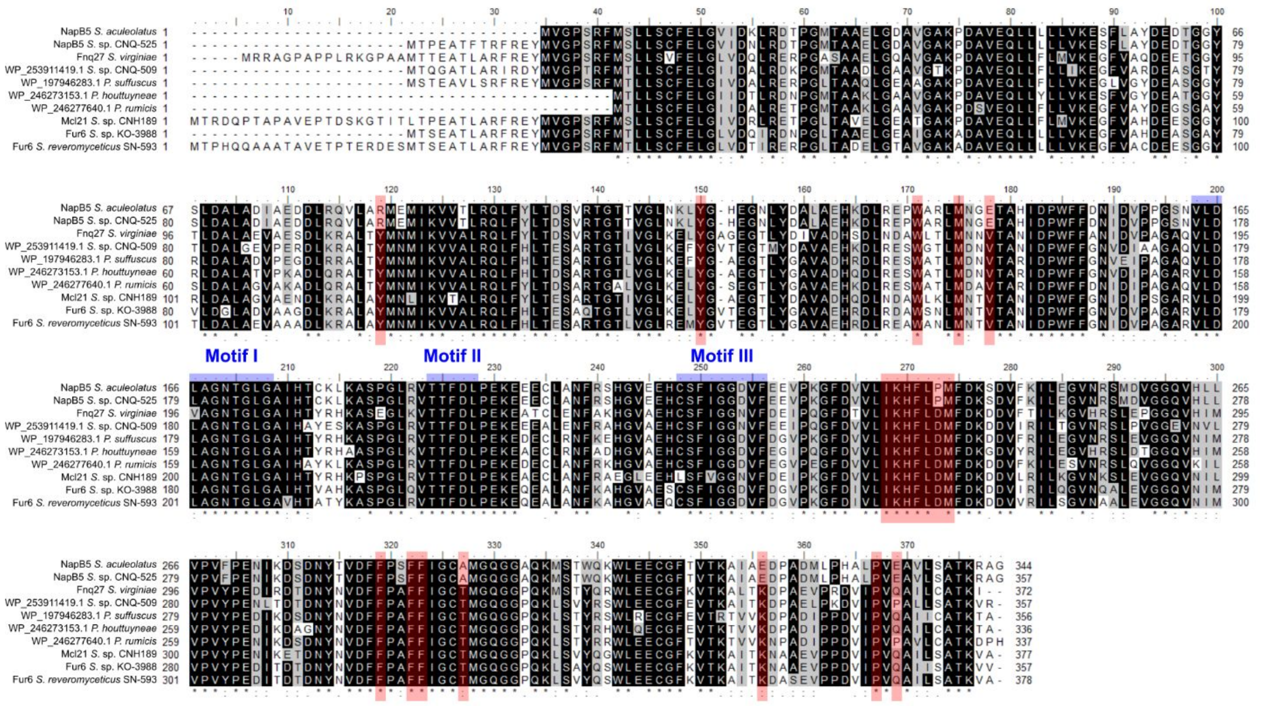


**Figure S2:** Multiple sequence alignment of C-MTs from $T_4HN$-derived meroterpenoid biosynthesis. Putative residues of the active sites are highlighted in red. Conserved SAM-binding motifs I–III are highlighted in blue.

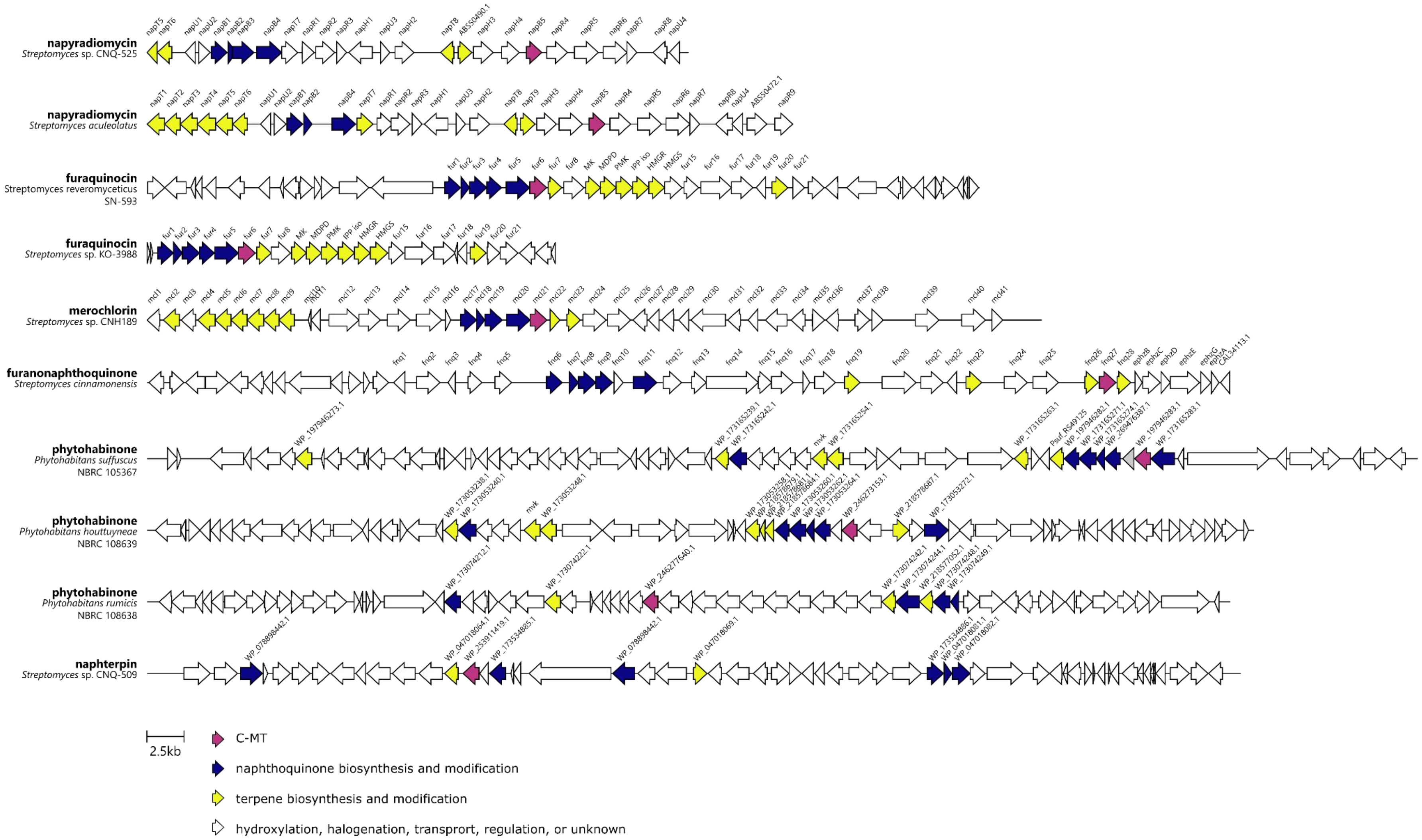


**Figure S3:** Biosynthetic gene cluster comparison of $T_4HN$-derived meroterpenoids from ascomycetes. Putative C-MT genes are highlighted in magenta.

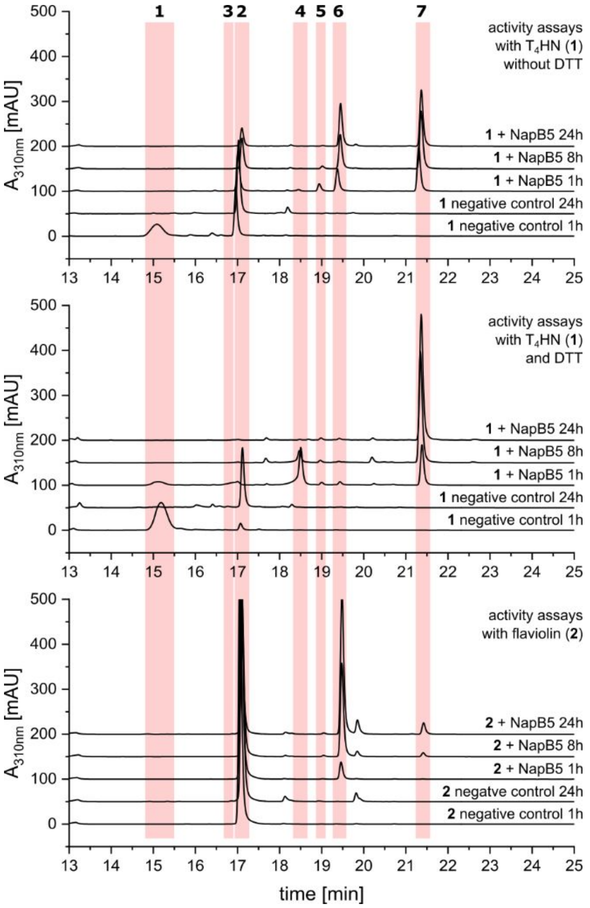


**Figure S4**: Comparison of HPLC-DAD chromatograms (310 nm) of NapB5 activity assays with **1**, with and without DTT, and **2**. The non-oxidized products **3** and **4** are only detectable when DTT is present. In the absence of DTT, **1** rapidly oxidizes to **2** leading to the main product **6** of **2** conversion. The assays were performed with purified enzyme.

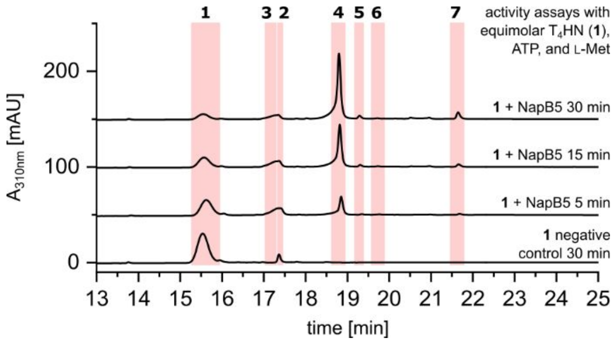


**Figure S5**: HPLC-DAD chromatograms (310 nm) of NapB5 activity assays with $T_4HN$ (**1**) and SAM precursors (ATP + L-methionine) at equimolar concentrations (0.5 mM each) extracted after 5, 15, and 30 minutes.

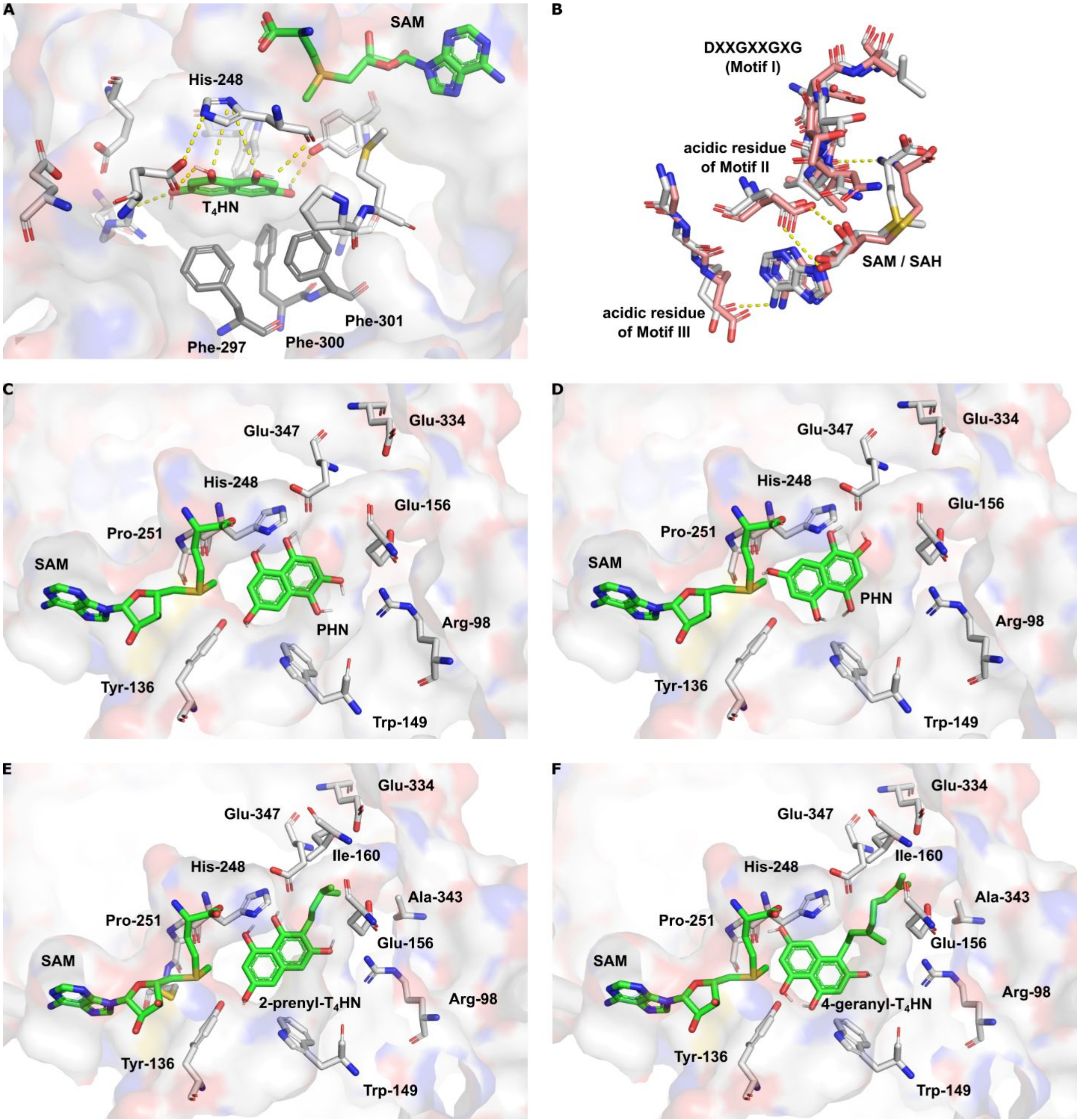


**Figure S6:** Docking studies of NapB5 model. **A**) Docking of SAM and $T_4HN$ (**1**). **B**) Comparison of SAM binding sites by superposition of NapB5 model docked with SAM (grey) onto the crystal structure of SAH-bound Fur6 (red, PDB: 8HAR). **C**) Docking of SAM and PHN (Conformation 1). **D**) Docking of SAM and PHN (Conformation 2). **E**) Docking of SAM and 2-prenyl-$T_4HN$. **F**) Docking of SAM and 4-geranyl-$T_4HN$, which is a proposed intermediate before C4-to-C3 α-hydroxyketone rearrangement of the geranyl moiety.[8] Ile-160 and Ala-343 may provide hydrophobic interactions with the prenyl/ geranyl chain.

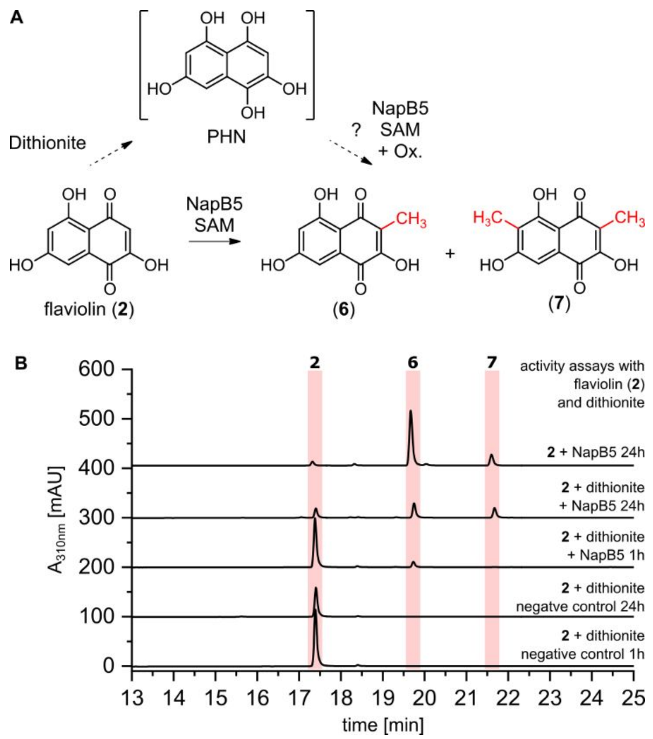


**Figure S7**: **A**) Reduction of flaviolin (**2**) to PHN in the presence of dithionite and conversion of flaviolin by NapB5. **B**) HPLC-DAD chromatograms (310 nm) of NapB5 activity assays with **2** and dithionite extracted after 1 or 24 h.

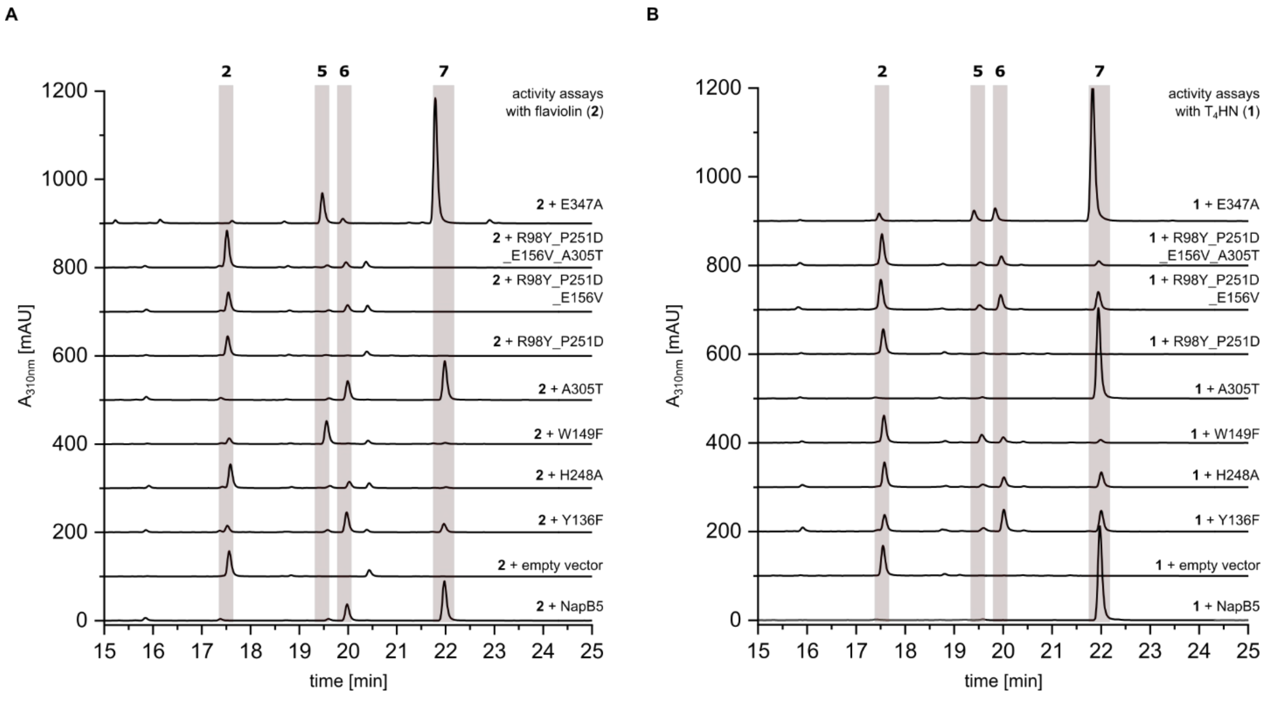


**Figure S8**: HPLC-DAD chromatograms (310 nm) of NapB5 wildtype and NapB5 variants activity assays with flaviolin (**2**) (**A**) and $T_4HN$ (**1**) (**B**) after 24h. The assays were performed with cell-free lysate of *E. coli* BL21Gold (DE3) cells transformed with the respective constructs. Cell-free lysate of *E. coli* BL21Gold (DE3) cells transformed with the empty vector pET28a was used for the negative control. Note that in cell-free lysate assays, $T_4HN$ (**1**) and its methylation products (**3** and **4**) undergo complete oxidation within 24 h.

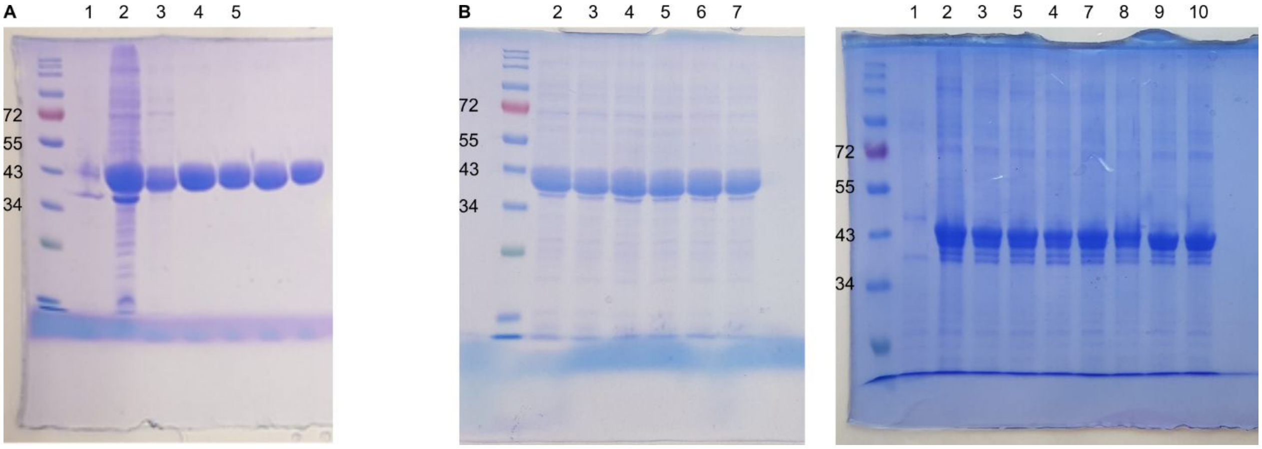


**Figure S9: A**) SDS-PAGE of His$_6$-NapB5 (43.2 kDa) Ni-NTA purification. Ladder: NEB Color Prestained Protein Standard. Fraction 1: cell pellet. 2: cell-free lysate. 3: 50 mM imidazole wash step. 4: 250 mM imidazole elution step. 5: desalted fraction. **B**) SDS-PAGE of cell-free lysate of His$_6$-NapB5 wildtype and variants (43.2 kDa). Fraction 1: *E. coli* BL21 Gold (DE3) cells transformed with pET28a empty vector. 2: NapB5 wildtype. 3: H248A. 4: Y136F. 5: W149F. 6: E347A. 7: A305T. 8 R98Y_P251D. 9: R98Y_P251D_E156V. 10: R98Y_P251D_E156V_A305T. The Coomassie blue staining shows comparable protein concentrations in wildtype and variant lysates.

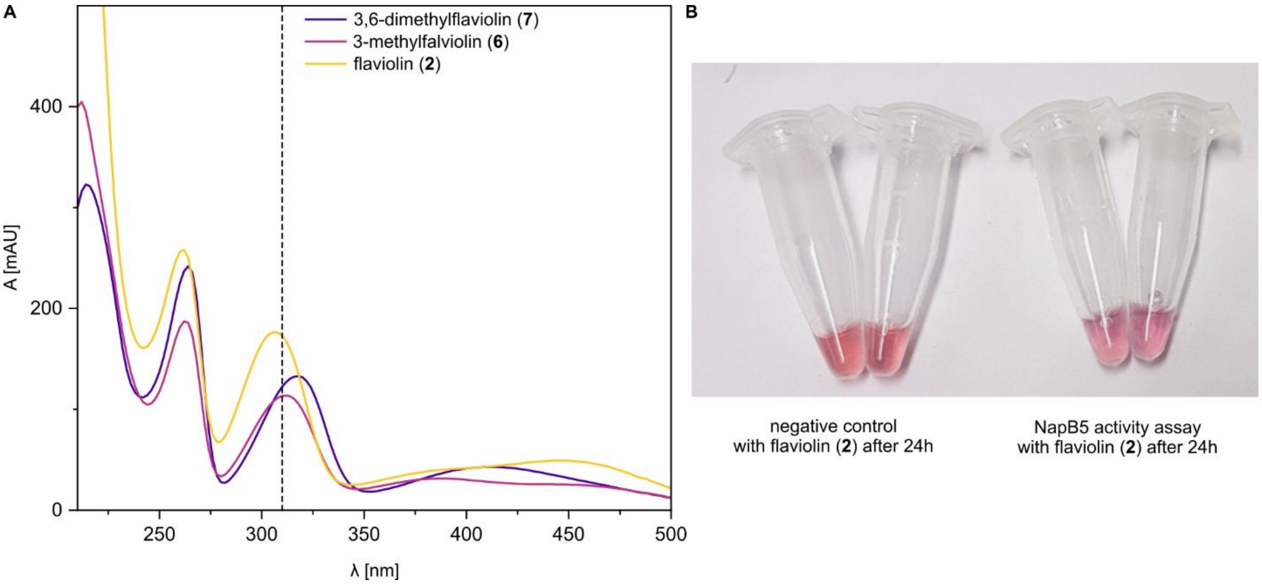


**Figure S10: A**) Extracted UV-spectra from HPLC-DAD peaks of flaviolin (**2**), 3-methylflaviolin (**6**), and 3,6-dimethylflaviolin (**7**). As a compromise for all assays, a wavelength of 310 nm (dashed line) was chosen to minimize the influence of the bathochromic shift on product formation calculations. **B**) Colors of NapB5 activity assays with **2**.

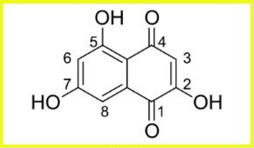

OH
O
5
4
6
3
7
2
HO
OH
8
1
O

(**2**)

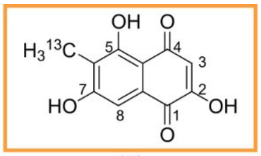

OH
O
$H_3{}^{13}C$
5
4
3
7
2
HO
OH
8
1
O

(**5**)

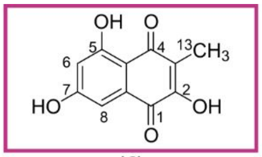

OH
O
5
4
$^{13}CH_3$
6
7
2
HO
OH
8
1
O

(**6**)

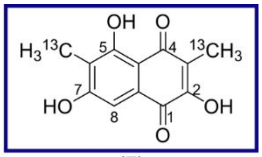

OH
O
$H_3{}^{13}C$
5
4
$^{13}CH_3$
7
2
HO
OH
8
1
O

(**7**)

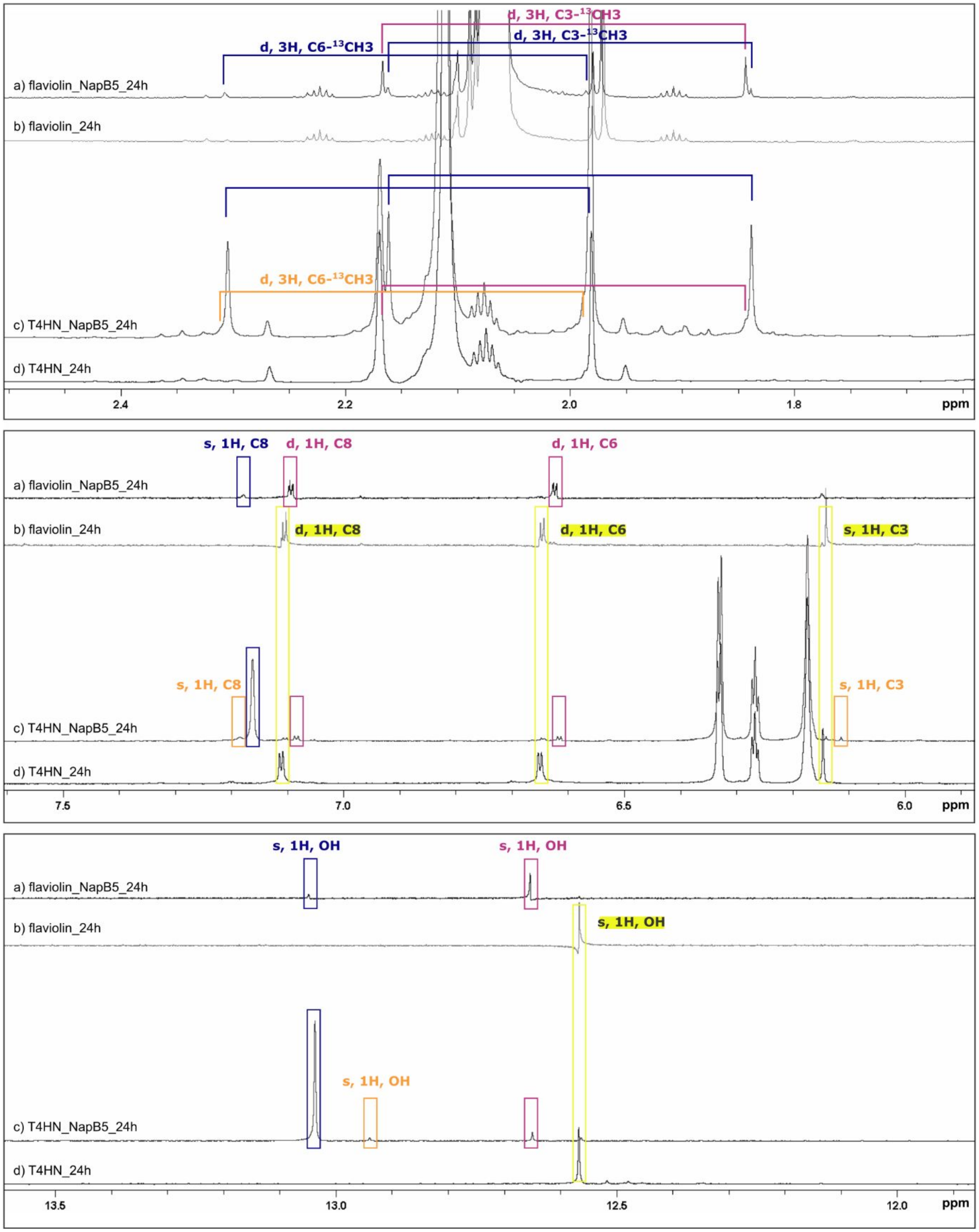

d, 3H, C3-13CH3
d, 3H, C3-13CH3
d, 3H, C6-13CH3
a) flaviolin_NapB5_24h
b) flaviolin_24h
d, 3H, C6-13CH3
c) T4HN_NapB5_24h
d) T4HN_24h
2.4
2.2
2.0
1.8
ppm
s, 1H, C8
d, 1H, C8
d, 1H, C6
a) flaviolin_NapB5_24h
b) flaviolin_24h
d, 1H, C8
d, 1H, C6
s, 1H, C3
s, 1H, C8
s, 1H, C3
c) T4HN_NapB5_24h
d) T4HN_24h
7.5
7.0
6.5
6.0
ppm
s, 1H, OH
s, 1H, OH
a) flaviolin_NapB5_24h
b) flaviolin_24h
s, 1H, OH
s, 1H, OH
c) T4HN_NapB5_24h
d) T4HN_24h
13.5
13.0
12.5
12.0
ppm

↑**Figure S11:** Comparison of $^{1}H$ NMR spectra (acetone-$d_6$) of extracted NapB5 activity assays of $T_4HN$ (**1**) or flaviolin (**2**) after 24h (trace c and a) and respective negative controls without enzyme (trace d and b). The signals corresponding to the respective compounds are color-coded. The protons of the $^{13}C$-labeled methyl groups appear as doublets with a heteronuclear coupling ($^{1}J_{CH}$) constant of 128 Hz. In NapB5 activity assays with **2**, signals of the main product **6** are highlighted in purple. These signals can also be found with lower intensity in assays with **1**. The main product of **1** conversion is **7** (blue) with the two methyl groups visible as doublets (128 Hz each) at 1.99 and 2.12 ppm, respectively. These signals can also be found with lower intensity in assays with **2**. Signals corresponding to **5** (orange) are only visible in assays with **1**. The doublet of the C6-methyl group is overlayed by the C6-methyl group of **7** (2.13 ppm for **5** and 2.12 ppm for **7**), but the aromatic protons of **5** are visible as singlets at 6.10 and 7.18 ppm, respectively.

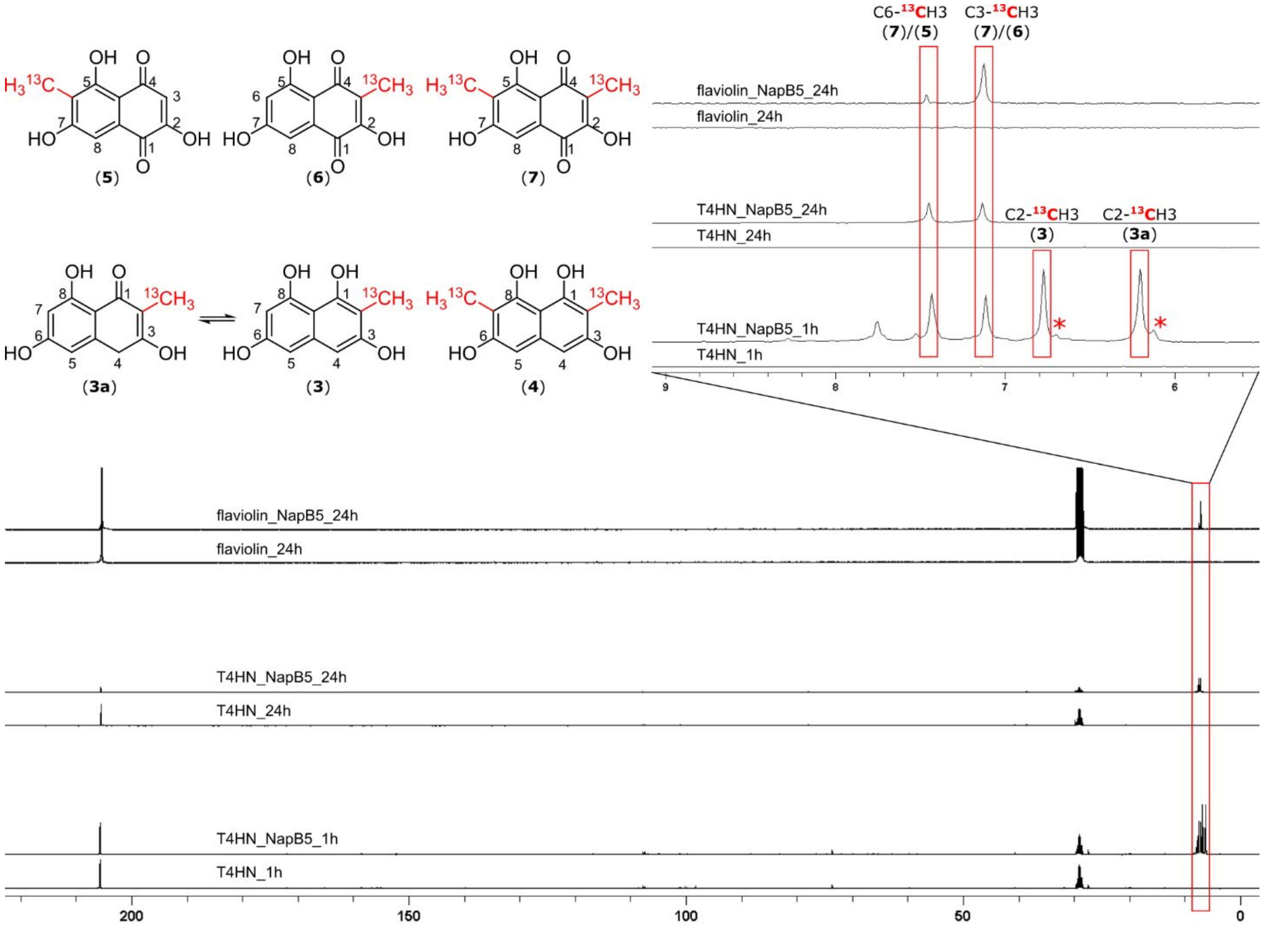


**Figure S12:** $^{13}C$ NMR spectra (acetone-$d_6$) of extracted NapB5 activity assays with $T_4HN$ (**1**) after 1 h and 24 h, with flaviolin (**2**) after 24 h, and respective negative controls without enzyme. Due to low substrate concentrations, only $^{13}C$-labeled methyl signals are visible. After 24 h, the $T_4HN$ (**1**) methylation products **3** and **4** are oxidized to **5**, **6**, and **7** with a signal at 7.44 ppm for the C6-methyl group for **5** and **7**, and 7.12 ppm for C3-methyl group for **6** and **7**. In spectra of 1h activity assays with **1** an additional signal at 6.77 ppm arises for the C2-methyl of **3** and at 6.20 ppm for the methyl group of the respective keto tautomer (**3a**). The small signals, marked with asterisks, may represent the methyl groups of the enol and keto tautomer of **4**. The signal at 7.75 ppm in the NapB5 activity assays with $T_4HN$ (**1**) after 1 h is not assigned because it does not clearly correlate with the $^{1}H$ spectrum in the HSQC spectrum (see Figure S10).

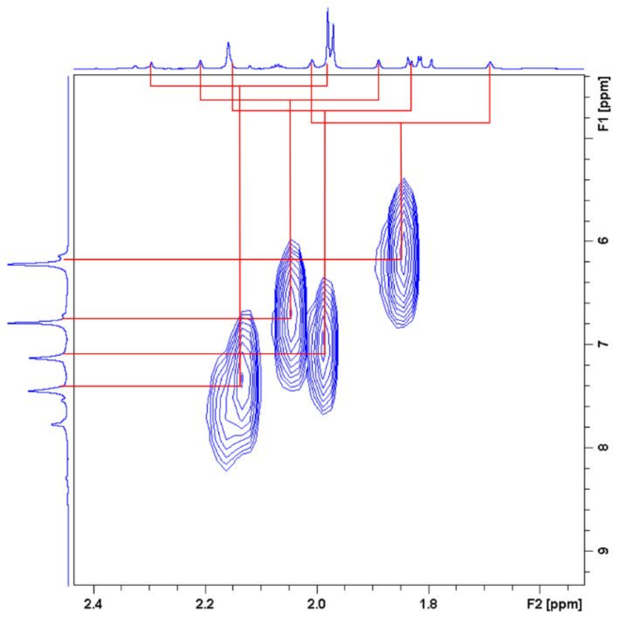


**Figure S13:** HSQC spectrum (acetone-$d_6$) of extracted NapB5 activity assay with $T_4HN$ (**1**) after 1 h. The $^{13}C$ signals of the labeled methyl groups correlate with the $^{1}J_{CH}$ doublets of the $^{1}H$ spectrum.

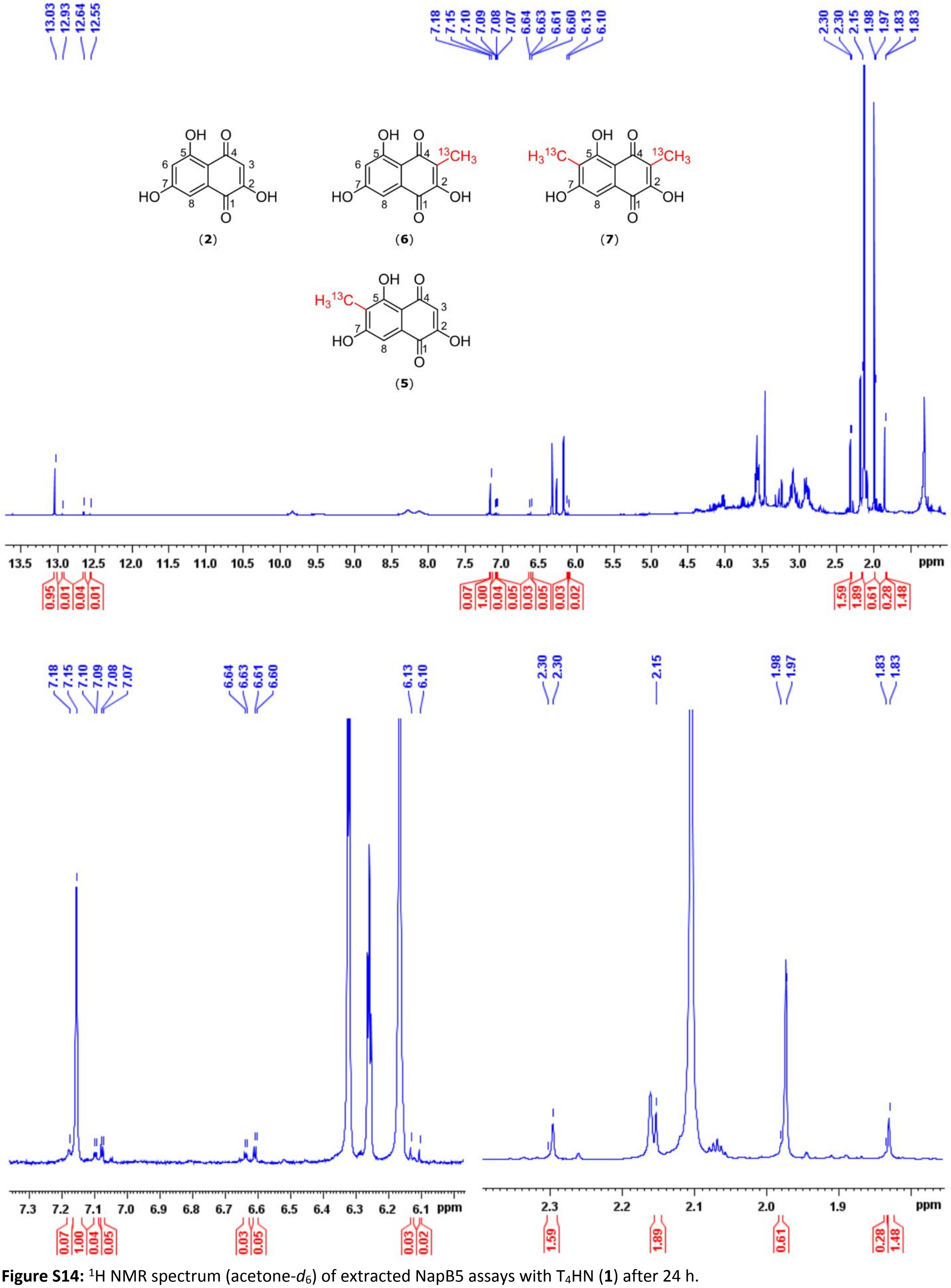


**Figure S14:** $^{1}H$ NMR spectrum (acetone-$d_6$) of extracted NapB5 assays with $T_4HN$ (**1**) after 24 h.

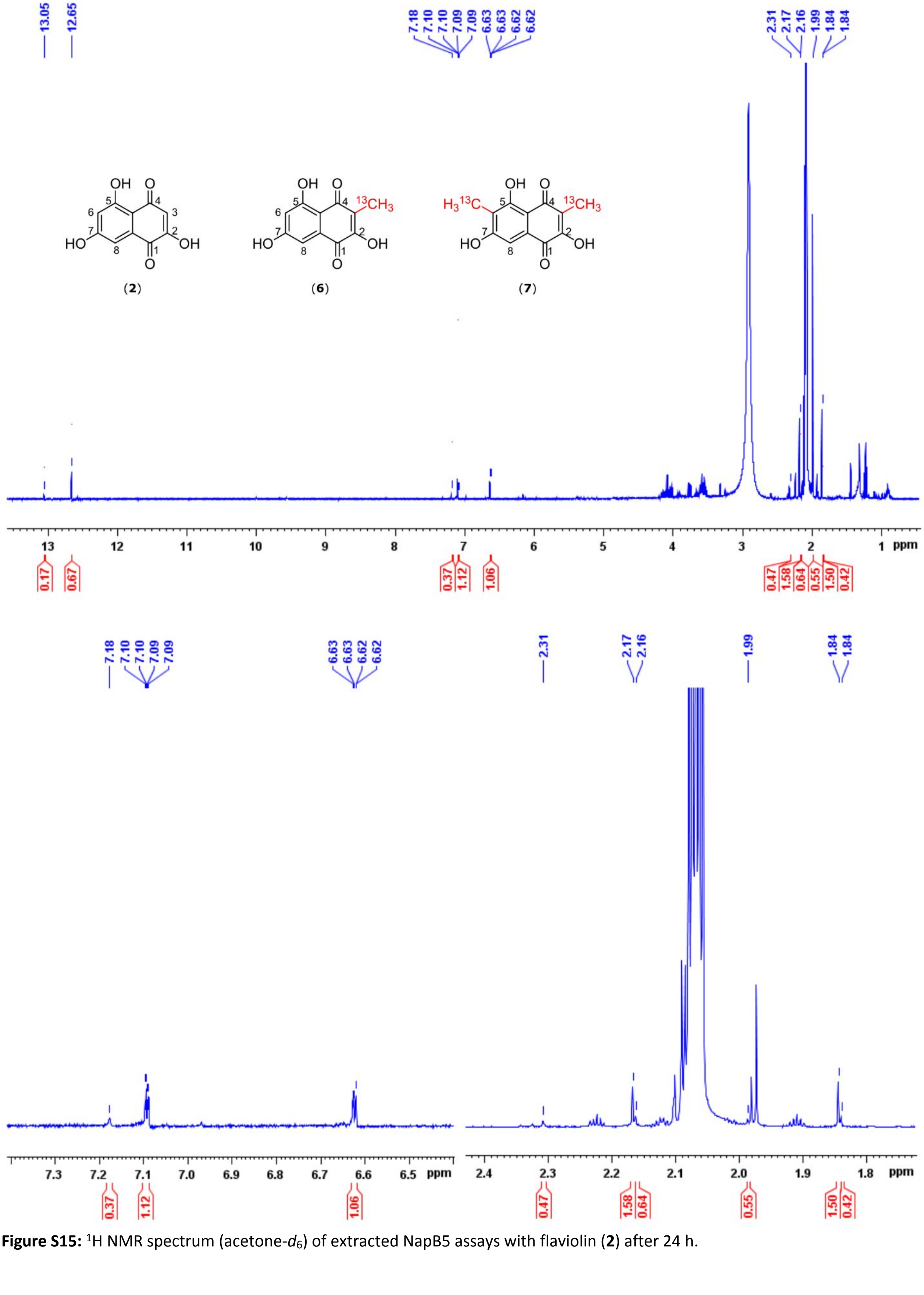


**Figure S15:** $^{1}H$ NMR spectrum (acetone-$d_6$) of extracted NapB5 assays with flaviolin (**2**) after 24 h.

**References:**

(1) Winter, J. M.; Moffitt, M. C.; Zazopoulos, E.; McAlpine, J. B.; Dorrestein, P. C.; Moore, B. S. Molecular Basis for Chloronium-mediated Meroterpene Cyclization: Cloning, Sequencing, and Heterologous Expression of the Napyradiomycin Biosynthetic Gene Cluster. *Journal of Biological Chemistry* **2007**, *282* (22), 16362–16368. DOI: 10.1074/jbc.M611046200.

(2) Panthee, S.; Takahashi, S.; Takagi, H.; Nogawa, T.; Oowada, E.; Uramoto, M.; Osada, H. Furaquinocins I and J: novel polyketide isoprenoid hybrid compounds from *Streptomyces reveromyceticus* SN-593. *Journal of Antibiotics* **2011**, *64* (7), 509–513. DOI: 10.1038/ja.2011.41.

(3) Kawasaki, T.; Hayashi, Y.; Kuzuyama, T.; Furihata, K.; Itoh, N.; Seto, H.; Dairi, T. Biosynthesis of a natural polyketide-isoprenoid hybrid compound, furaquinocin A: identification and heterologous expression of the gene cluster. *Journal of Bacteriology* **2006**, *188* (4), 1236–1244. DOI: 10.1128/JB.188.4.1236-1244.2006.

(4) Kaysser, L.; Bernhardt, P.; Nam, S.-J.; Loesgen, S.; Ruby, J. G.; Skewes-Cox, P.; Jensen, P. R.; Fenical, W.; Moore, B. S. Merochlorins A-D, cyclic meroterpenoid antibiotics biosynthesized in divergent pathways with vanadium-dependent chloroperoxidases. *Journal of the American Chemical Society* **2012**, *134* (29), 11988–11991. DOI: 10.1021/ja305665f.

(5) Haagen, Y.; Glück, K.; Fay, K.; Kammerer, B.; Gust, B.; Heide, L. A gene cluster for prenylated naphthoquinone and prenylated phenazine biosynthesis in *Streptomyces cinnamonensis* DSM 1042. *ChemBioChem* **2006**, *7* (12), 2016–2027. DOI: 10.1002/cbic.200600338.

(6) Kuzuyama, T.; Noel, J. P.; Richard, S. B. Structural basis for the promiscuous biosynthetic prenylation of aromatic natural products. *Nature* **2005**, *435* (7044), 983–987. DOI: 10.1038/nature03668.

(7) Noguchi, T. 放線菌のメロテルペノイド生合成に見出された 新規窒素代謝に関する研究. PhD Thesis, The University of Tokyo, 2022.

(8) McKinnie, S. M. K.; Miles, Z. D.; Jordan, P. A.; Awakawa, T.; Pepper, H. P.; Murray, L. A. M.; George, J. H.; Moore, B. S. Total Enzyme Syntheses of Napyradiomycins A1 and B1. *Journal of the American Chemical Society* **2018**, *140* (51), 17840–17845. DOI: 10.1021/jacs.8b10134.
